# Insight into the Varying Reactivity of Different Catalysts for CO_2_ Cycloaddition into Styrene Oxide: An Experimental and DFT Study

**DOI:** 10.3390/ijms24032123

**Published:** 2023-01-20

**Authors:** Angelo Pio Sebaaly, Hugo Dias, Lorraine Christ, Lynda Merzoud, Henry Chermette, Guillaume Hoffmann, Christophe Morell

**Affiliations:** 1 Institut des Sciences Analytiques UMR CNRS 5280, Université Claude Bernard Lyon1, Université de Lyon, 5 Rue La Doua, 69622 Villeurbanne, France; 2 Institut de Recherches sur la Catalyse et l’Environnement de Lyon, IRCELYON, UMR CNRS 5256, Université Claude Bernard Lyon1, Université de Lyon, 2 Av. Albert Einstein, 69626 Villeurbanne, France

**Keywords:** DFT, non-covalent interactions, catalysis, CO_2_ capture, mechanistic study

## Abstract

The cycloaddition of CO_2_ into epoxides to form cyclic carbonates is a highly sought-after reaction for its potential to both reduce and use CO_2_, which is a greenhouse gas. In this paper, we present experimental and theoretical studies and a mechanistic approach for three catalytic systems. First, as Lewis base catalysts, imidazole and its derivatives, then as a Lewis acid catalyst, ZnI_2_ alone, and after that, the combined system of ZnI_2_ and imidazole. In the former, we aimed to discover the reasons for the varied reactivities of five Lewis base catalysts. Furthermore, we succeeded in reproducing the experimental results and trends using DFT. To add, we emphasized the importance of non-covalent interactions and their role in reactivity. In our case, the presence of a hydrogen bond was a key factor in decreasing the reactivity of some catalysts, thus leading to lower conversion rates. Finally, mechanistically understanding this 100% atom economy reaction can aid experimental chemists in designing better and more efficient catalytic systems.

## 1. Introduction

**Scheme 1:**
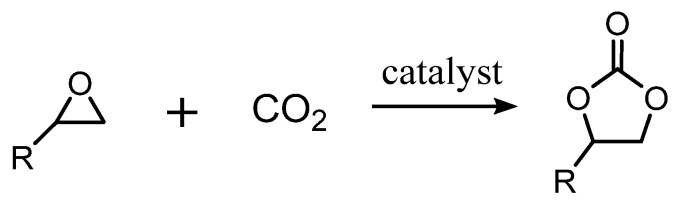
CO_2_ reaction with epoxides giving cyclic carbonates.

Climate change, which is being exacerbated by current global warming, has emerged as one of the century’s most pressing concerns. In the scientific community, we can retrace and find the first published evidence from Callendar in 1938 who demonstrated that the climate was warming and CO_2_ levels were rising [1]. Carbon dioxide (CO_2_) is a greenhouse gas that absorbs infrared radiation that is emitted from Earth and that happen to cool its surface after a day in the Sun. This radiation, which is supposed to escape the atmosphere, is trapped by CO_2_ [2]. In consequence, dire and sometimes irreversible effects are observed all around the world [3]. Many efforts have been dedicated to mitigate this global issue over the last decades. However, it was only until the late 1970s that the idea of Carbon dioxide Capture and Storage (CCS) was proposed as a solution to tackle the CO_2_ issue [4,5]. Furthermore, the Intergovernmental Panel on Climate Change (IPCC) issued a report in 2005 that discussed this specific issue and laid a substantial part of the framework for scientists today [6]. Since then, CO_2_ was considered as a carbon source for chemical reactions, consequently, several chemical routes for CCS have been proposed and studied, among which was the use of carbon dioxide as a reactant in organic synthesis, and the incorporation of CO_2_ ultimately forming organic carbonates [7,8,9]. In this paper, we will be focusing on the latter, more specifically, the conversion of epoxides into cyclic carbonates via CO_2_ cycloaddition (Scheme 1).

**Figure 1:**
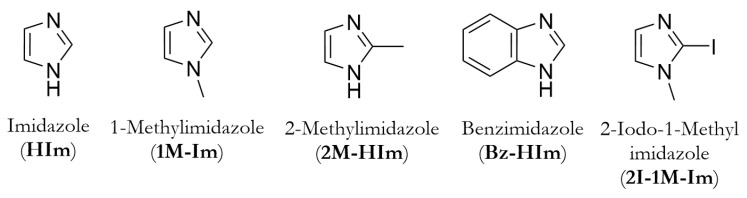
Imidazole and four of its derivatives.

This conversion has become quite popular over the years for the conversion of captured CO_2_ (from, for example, waste combustion, flue gases or in the atmosphere) into useful products. Indeed, cyclic carbonates have been shown to have numerous applications, and they can be used as solvents for organic reactions [10], as monomers for polymerization [11,12,13,14], as well as reactive agents in numerous synthetic pathways [15,16,17]. This reaction can be accelerated with the use of some catalysts, the first of which was a polymer-supported nanogold catalyst [18]. Numerous parallels between these various catalytic systems have been discovered, which has led researchers to the conclusion that the catalyst must adhere to certain criteria in order to effectively catalyze the reaction. The catalytic system must contain for example, a nucleophile/Lewis base that should be a good leaving group such as halides or N-heterocycles [19,20]. It should also include Lewis acid sites that can be found in metals for example [19,21], or from a theoretical point of view, Brønsted acid sites able to donate protons, which are the simplest Lewis acids. Lastly, it is important to mention that there are reports in the importance of hydrogen bond donor sites [22]. In this article we will study Imidazole (HIm) and four of its derivatives: 1-Methylimidazole (1M-Im), 2-Methylimidazole (2M-HIm), Benzimidazole (Bz-HIm) and 2-Iodo-1-Methylimidazole (2I-1M-Im) as Lewis Base catalysts (Figure 1).

The purpose of this study is not only to unravel the mechanism of the CO_2_ cycloaddition, but also to try to gain some insight into the reasons associated with the increase in activity, and in doing so, optimizing this catalytic reaction using quantum calculations.

## 2. Results and Discussion

### 2.1. Experimental Results

The catalytic activity of these compounds for the cycloaddition of CO_2_ were measured in solvent-free conditions with Styrene Oxide (SO) as the epoxide. SO is a stable well-known substrate often used in catalytic studies [23,24]. Various imidazole derivates or ZnI_2_ or their combination were used in 1.0 mol% vs. epoxide at 100 °C, during 18 h under 2.0 MPa of CO_2_. Styrene oxide conversion and cyclic Styrene Carbonate (SC) yield were determined by ^1^H NMR spectroscopy using an internal standard. All the main experimental data are compiled in Table 1.

All imidazole and its derivatives (entries 1–5) are able to catalyze the reaction between CO_2_ and styrene oxide to selectively form cyclic styrene carbonate, even if moderate yields are obtained (31–53%), except for 2-Iodo-1-Methylimidazole (entry 5). In the case of ZnI_2_ (entry 6), a low carbonate yield was obtained despite a high epoxide conversion. This lack of selectivity is largely improved when combining the zinc salt to any of the imidazole compounds in equimolar ratios, as for example HIm (entry 7) leading to almost complete SO conversion and 98% SC yield. To compare our results with experimental data, styrene oxide was also chosen as the epoxide for our theoretical calculations. This trend was also noticed when combining ZnBr_2_ as Lewis acid with HIm as Lewis base [25]. The use of Zn complexes to catalyze the insertion of CO_2_ into epoxides, in particular with salphen ligand, has been the object of several studies in the past [26,27,28].

### 2.2. Theoretical Results and Discussion

To compare our results with experimental data, styrene oxide was chosen as the epoxide for the theoretical calculations. The results will be divided into three main parts. In the first section, the reaction mechanism of imidazole and its derivatives without the presence of ZnI_2_ will be discussed. An insightful discussion on the catalysts performance is included through the pathway with a Lewis Base (denoted LB throughout the article). In the second and third sections, the complete reaction pathway pertaining to the ZnI_2_ catalyzed and Lewis acid (ZnI_2_) + Lewis base (HIm) catalyzed reactions are discussed (denoted LA in this section). Theoretical calculations and fruitful results using the NCI (Non Covalent Index) are presented. At last, a concise and precise description of the experimental results, their interpretation, as well as the experimental conclusions are provided.

#### 2.2.1. Lewis Base Mechanism

**Scheme 2:**
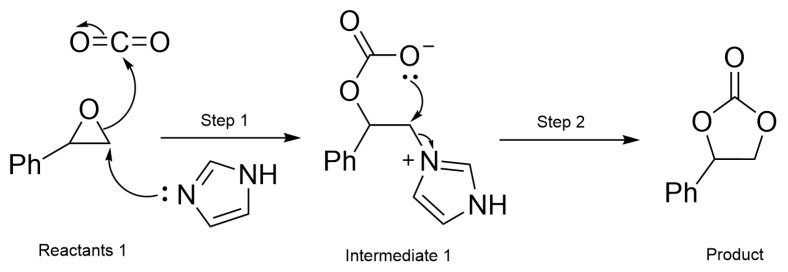
Proposed reaction mechanism for the Lewis base catalysed cycloaddition of CO_2_ into SO to form SC. Imidazole is taken as representative example for all the Lewis base catalysts of this study.

The pathway we propose is a two-step concerted mechanism (Scheme 2), similar to the one presented by Roshan et al. [20]. The first step is the attack of the nucleophile (catalyst) on the less-substituted carbon of the epoxide, and the opening of the latter. Simultaneously, the epoxide oxygen attacks the electrophilic carbon of CO_2_ leading to Intermediate **1**. The second and final step is an SN2 reaction where the free oxygen plays the role of the nucleophile closing the ring, and releasing the catalyst which then plays the role of a leaving group to produce SC.

**Figure 2:**
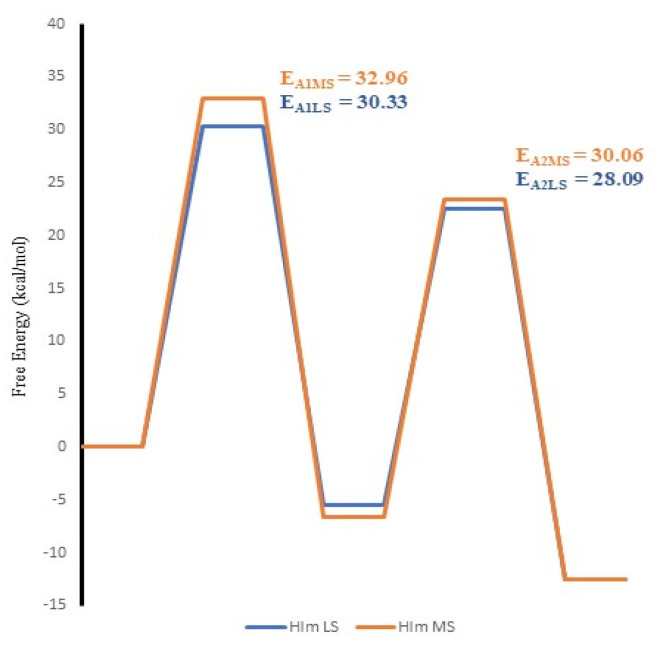
Free energy diagrams (kcal/mol) of HIm catalysed CO_2_ cycloaddition, proceeding from the attack of the LB on the more substituted side of the epoxide in orange (HIm MS), in addition to that proceeding from the attack from the less substituted side in blue (HIm LS).

**Figure 3:**
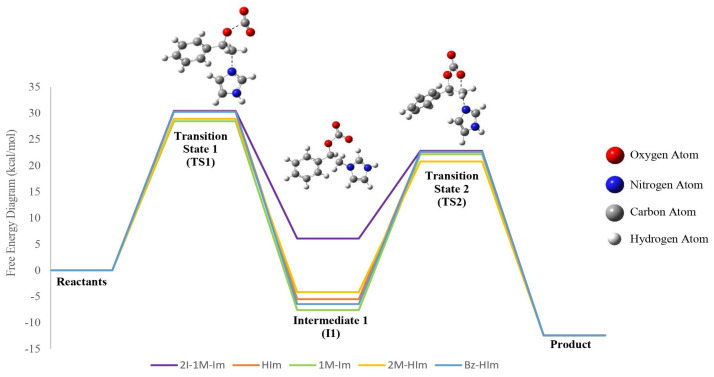
Free energy diagram (kcal/mol) reaction **1** with different catalysts; Imidazole (orange), 1-Methylimidazole (green), 2-Methylimidazole (yellow), Benzimidazole (blue), and 2-Iodo-1-Methylimidazole (purple). HIm has been selected as the representative Lewis base for structure representation.

To explain the regioslectivity of the first step, we have computed free energy diagrams for both possible pathways. The results are presented in Figure 2. As shown in this figure, both activation energies pertaining to the Least Substituted (LS) pathway being 30.33 kcal/mol and 28.09 kcal/mol for E_A1LS_ and E_A2LS_, respectively, are slightly less than those for the More Substituted (MS) pathway, which are 32.96 kcal/mol and 30.06 kcal/mol for E_A1MS_ and E_A2MS_, respectively. Given that the rate determining step, the LS pathway is slightly more favorable than the MS pathway. However, the difference of less than 2 kcal/mol implies that both pathways are in competition. Similar results are seen in all the other LB catalysts, and their free energy diagrams can be found in the Supplementary Materials (Figures S1–S4). Following this, the free energy diagram showing a comparative view of the different pathways for all the different catalysts in kcal/mol is presented in Figure 3. Table 2 shows the activation energies for the 1st step and the 2nd step as E_A1_ and E_A2_, respectively for all the catalysts.

The first thing to note is the small differences in E_A1_, the range being 2 kcal/mol. From this, and taking into consideration a margin of error for the calculations, it can be concluded that the first step is nearly equivalent for all LBs. To put it more clearly, varying the catalyst does not markedly change the energy of the first transition state.

In contrast, the differences in E_A2_ are more prominent, with a range of more than 13 kcal/mol. This is largely due to the free energy of the intermediate I1. Interestingly, I1 of the yellow curve belonging to the 2M-HIm LB, is considerably less stable than those of HIm, 1M-Im, and Bz-HIm. Additionally, the intermediate pertaining to the 2I-1M-Im pathway is the highest in energy by far and therefore the most reactive among the intermediates. While analyzing the optimized structures of all intermediates, we noticed that the reason for the considerable differences in stability lies in the intermolecular interactions between the atom/substituent on carbon 2 of the imidazole catalyst (the carbon atom between the two nitrogen atoms), and the free nucleophilic oxygen. Consequently, we calculated the intramolecular interactions using the Non-Covalent Interaction (NCI) method [29] in this particular region. 

**Figure 4:**
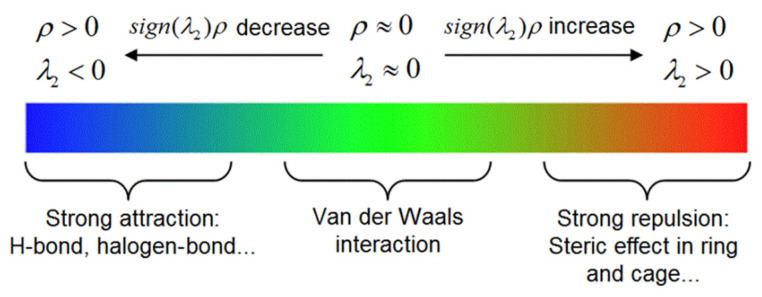
Color-coded legend explaining the significance of sign (λ_2_)ρ to help interpret the scatter graph.

**Figure 5:**
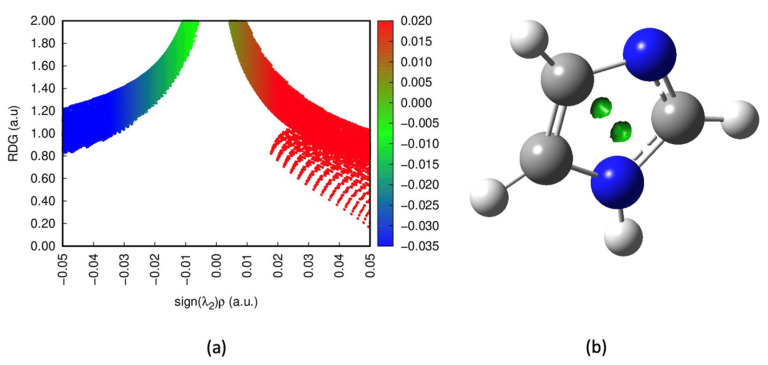
(**a**) RDG scatter graph for imidazole. (**b**) Visual representation of NCI interactions in imidazole (green).

**Figure 6:**
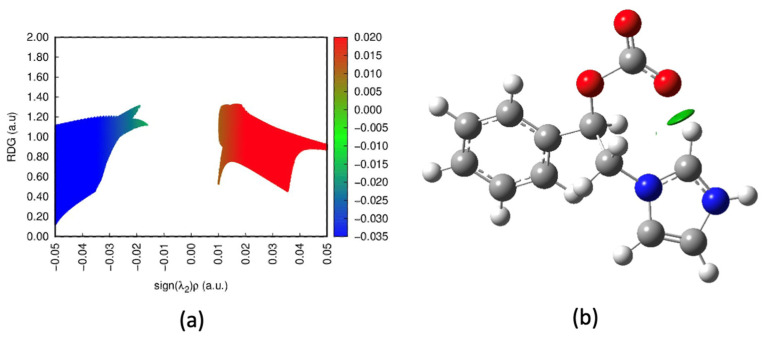
(**a**) The Reduced Density Gradient (RDG) scatter graph representing the NCI of I1 HIm shown in 6b. (**b**) Visual representation of the NCI as a green surface.

**Figure 7:**
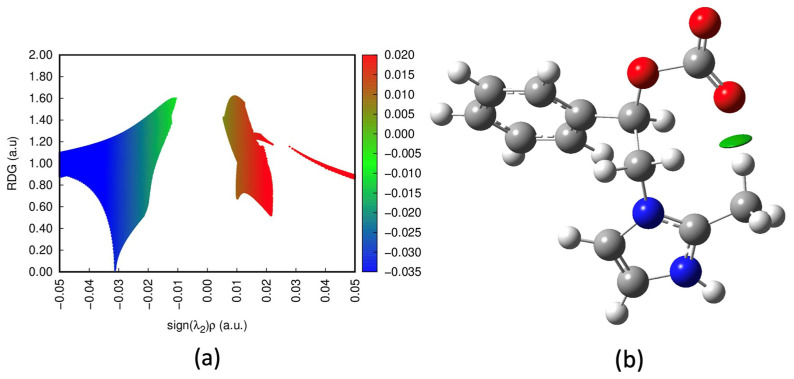
(**a**) The Reduced Density Gradient (RDG) scatter graph representing the NCI of I1 2M-HIm shown in Figure 7b. (**b**) Visual representation of the NCI as a green surface.

**Figure 8:**
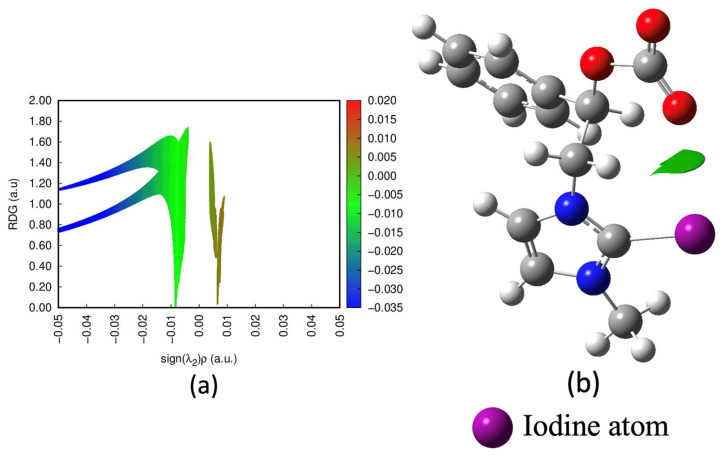
(**a**) The Reduced Density Gradient (RDG) scatter graph representing the NCI of **I1** 2I-1M-HIm shown in Figure 8b. (**b**) Visual representation of the NCI as a green surface.

The Reduced Density Gradient (RDG) calculated within the NCI methodology is a great tool to characterize the nature of the Non-Covalent Interaction in a particular region. To help read the scatter graphs, a color-coded legend is represented in Figure 4 [30]. The RDG (*y*-axis) is a dimensionless quantity that approaches 0 when there are covalent and/or non-covalent interactions. For a clearer picture, we used a range of sign (λ_2_)ρ that describes only weak interactions such as hydrogen bonds, Van der Waals interactions and steric repulsion (Figure 4). In the case of an absence of a distinguished peak with an RDG value close to 0, as for example in Figure 5, we can assume that no NCI are present. Figure 5 shows the optimized structure and the RDG scatter graph for a single imidazole molecule. This graph allows further comparison with the catalyst structures that are related. Furthermore, in Figure 6, Figure 7 and Figure 8, we show the surfaces of the intramolecular interactions of I1 HIm, 2M-HIm and 2I-1M-Im, respectively, as well as their RDG scatter graph. The NCI graph and results for I1 1M-Im and Bz-HIm are present in the Supporting Information (Figures S5 and S6).

When comparing Figure 5a with Figure 6a, we can see a clear blue peak upon extrapolation with a sign (λ_2_)ρ < −0.05 a.u. indicating the presence of a strong hydrogen bond represented by the green surface in Figure 6b, which represents the NCI of I1 HIm.

Regarding Figure 7, which represents I1 2M-HIm, we can see a blue peak just below sign (λ_2_)ρ = −0.03 a.u. which similar to I1 HIm, shows the presence of a hydrogen bond (Figure 7a). However, from the sign (λ_2_)ρ value, we can deduce that the strength of that H-bond is weaker than that in Figure 6a. It can also be seen that the presence of a methyl group in position 2 of the Lewis base catalyst results in a weaker interaction.

Moreover, this accounts for a more reactive intermediate and a smaller E_A2_. Finally, Figure 8a shows complete eradication of the H-bond as it can be shown with an absence of any blue peak. Instead, we can see a green peak at sign (λ_2_)ρ slightly higher than −0.01 a.u. demonstrating a Van der Waals interaction. In addition, a light green peak is clearly observed around 0.008 a.u. which is indicative in an NCI context of some steric effects. The visual representation of these interactions is shown as a green surface in Figure 8b. The trend of yield (%) is inversely proportional to the strength of the non-covalent interactions present in Intermediate 1 and to E_A2_.

#### 2.2.2. Lewis Acid Mechanism

**Figure 9:**
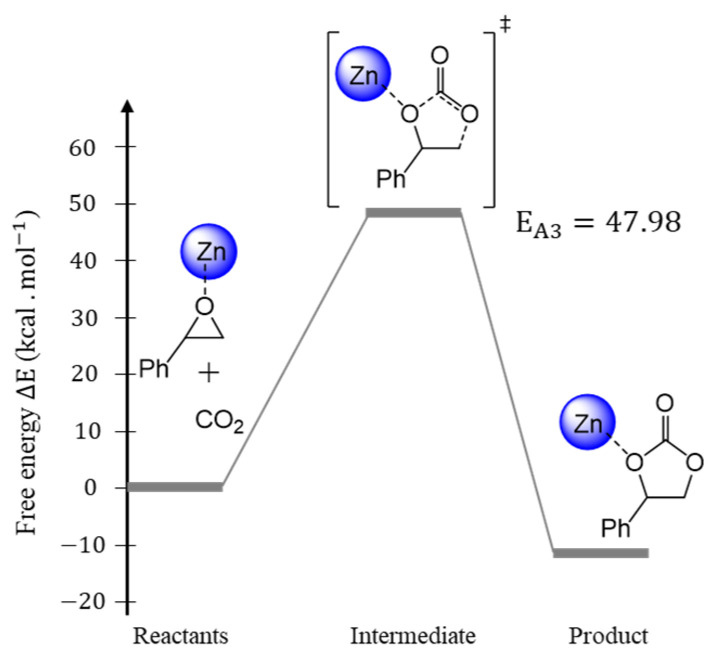
Free energy diagram (kcal/mol) of ZnI_2_ catalyzed transformation of SO to SC.

The LA catalyzed pathway follows a one-step concerted mechanism. The opening of the epoxide ring and the cycloaddition of the carbon dioxide are performed simultaneously. The free energy diagram, along with the structure of the transition state are presented in Figure 9. The activation energy (noted E_A3_ here) for this transformation is higher than those of the LB-catalyzed mechanism with a value of 48 kcal/mol. This high barrier gives rise to the possibility of competitive reactions and can explain the low yield of 21% obtained experimentally (Table 1).

#### 2.2.3. Lewis Acid Mechanism in Presence of ZnI_2_

The third part of the discussion will be around the effect of the addition of ZnI_2_ as a Lewis acid co-catalyst for this reaction. The results in Table 1 indicate a 45% yield with HIm alone, a 21% yield with ZnI_2_ as the sole catalyst, and a 98% yield when both are combined. The higher yield in entry 7 has led us to conclude that the mechanism involves a synergistic effect of both catalysts rather than a cumulative effect.

**Figure 10:**
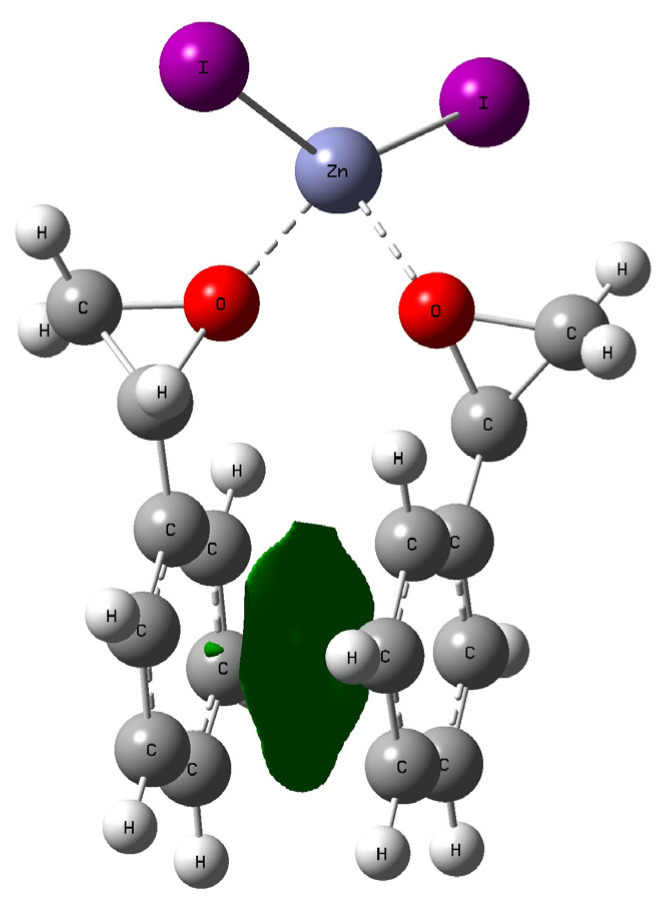
Proposed tetrahedral ZnI_2_(SO)_2_ complex with a π-π interactions (green).

The first suggestion would be that Zinc (II) complexes are usually four-coordinate complexes and take a tetrahedral or distorted tetrahedral geometry [31,32,33]. Some studies also show the possibility of a five-coordinate complex with a trigonal bipyramidal geometry; however, these instances are rare in the literature, and require certain conditions to be met [34,35,36,37]. Another possibility we ruled out is a polynuclear Zn catalyst because no condition is met for such structure, ZnI_2′_s catalytic concentration being only 1.0 mol%. In order to predict ZnI_2′_s dissociation, we calculated theoretically the Zn-I Bond Dissociation Energy (BDE) using the same level of theory as precedent. Results showed a BDE of dissociating the first iodide ion to be 192.75 kcal/mol. This high BDE value revealed that both iodide ions would remain attached to the zinc atom. Indeed, several studies indicate the robustness of Zn-I bonds upon addition of N-heterocycles [38,39,40,41]. Taking all of this into consideration, we propose a mononuclear, tetrahedral ZnI_2_(SO)_2_ complex presented in Figure 10. We can see in the latter that this Zn atom adopts a tetrahedral geometry ionically bonded to two iodide ions and linked with two styrene oxide with coordination bonds. Each of these SO ligands interact with each other via an NCI π-π interactions (represented with the green surface).

**Scheme 3:**
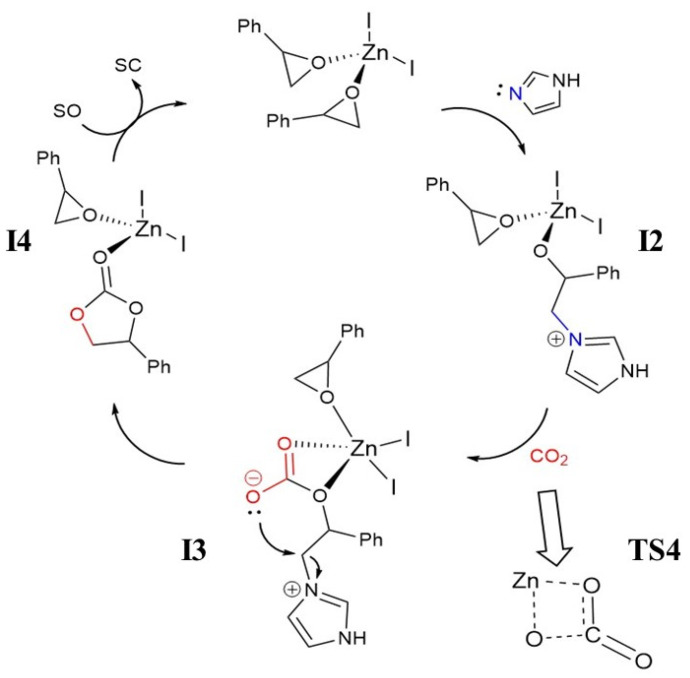
Proposed reaction mechanism for the HIm/ZnI_2_ catalyzed cycloaddition.

Using this structure, we propose a mechanistic pathway for the transformation of the SO into SC (Scheme 3). The first step being the attack of the imidazole on the epoxide, opening the epoxide and forming Intermediate 2 (I2). In the following step, the CO_2_ inserts itself via a four-membered cycle transition state (TS4). This leads to a penta-coordinated trigonal bipyramidal intermediate (I3). After that, ring closing occurs in an SN_2_ type mechanism where the free oxygen acts as a nucleophile and the Imidazolium as the leaving group leading to Intermediate 4 (I4). The final step is the substitution of the newly formed SC by a SO to regenerate the catalyst.

**Figure 11:**
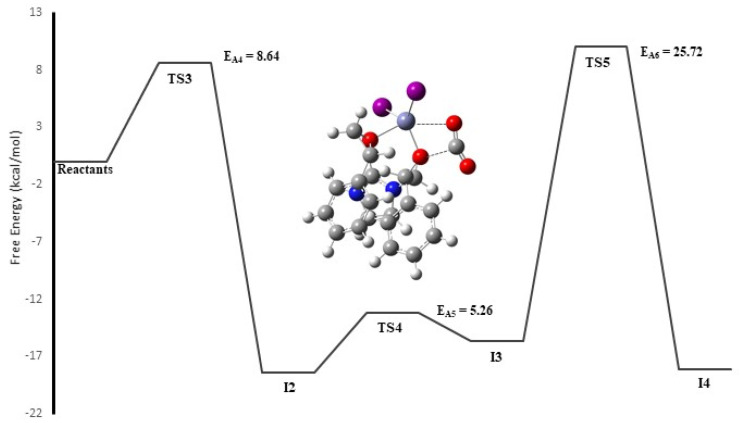
Free energy diagram (kcal/mol) of HIm/ZnI_2_ catalyzed transformation of styrene oxide (SO) to styrene carbonate (SC).

Figure 11 shows the free energy diagram for a potential mechanism of the LA + LB catalyzed pathway. It is important to note the relatively lower activation energy of the epoxide ring opening step (E_A4_ = 8.64 kcal/mol) compared to the LB catalyzed mechanism (E_A1HIm_ = 30.33 kcal/mol). The relatively low energies of TS2 and TS3 (E_A5_ = 5.26 kcal/mol) are also noteworthy. The rate-determining step in this reaction is the ring closure with an activation energy of E_A6_ = 25.72 kcal/mol. Comparing this value with the activation energies of the rate-determining steps of the previous catalytic systems, E_A6_ is the lowest. These theoretical results are in accordance with the experimental results when looking at entries 1, 6, and 7 (Table 1).

## 3. Materials and Methods

### 3.1. Computational Details

Styrene oxide was chosen as the epoxide for all the reactions considered in this study alongside CO_2_. The following Lewis bases were selected as catalysts; Imidazole (HIm), 1-Methylimidazole (1M-Im), 2-Methylimidazole (2M-HIm), Benzimidazole (Bz-HIm) and 2-Iodo-1-Methylimidazole (2I-1M-Im); finally ZnI_2_ was selected as the Lewis acid.

All geometry optimizations and energy calculations were performed using Density Functional Theory (DFT). The functional used in this work is ωB97X-D [42], mainly because it is a range-separated functional, which can capture both short and long-range interactions. The basis set chosen for all the calculations was LANL2DZ [43,44,45], which is a widely used basis set for systems containing heavy atoms, with a double equality and a relativistic effective core potential for iodide. All calculations have been performed in gas phase. Frequencies were computed in order to verify that no imaginary frequencies are present for the reactants, intermediates, and products. All transition states used in this work have only one imaginary frequency. Intrinsic Reaction Coordinate (IRC) calculations were performed on all transition states with the same level of theory to make sure that the transition structure leads to the sought-after reactants and products. Furthermore, all the free energies were calculated at 298.15 K, 1 atm. All the optimized structures were calculated using Gaussian09 [46] and Gaussian16 [47] quantum packages.

In all the free energy diagrams, the reactants are taken as reference with a ΔG = 0 kcal/mol.

Concerning the characterization and visualization of the non-covalent interactions, the approach used was that which was introduced by Johnson et al. [29] in 2010. Finally, the visualization of the RDG scatter graphs in a mapped color code representing the NCIs was made possible by Multiwfn [48] using the Independent Gradient Model [30].

### 3.2. Experimental Details

#### 3.2.1. General Considerations

All chemicals and some solvents were purchased from Sigma-Aldrich^®^ (St. Louis, MO, USA) and used as received, in particular styrene oxide (97% purity), imidazole (99% purity) and ZnI_2_ (98% purity). Carbon dioxide (99.995 % purity) was supplied by Air Liquide. ^1^H NMR spectra in CDCl_3_ were acquired on a Bruker Ascend™ 400 spectrometer (Bruker Corporation^®^, Billerica, MA, USA) at 298 K and referenced to the solvent signals.

#### 3.2.2. Catalytic Tests

All the catalytic tests were performed in a 40 mL stainless steel reactor equipped with a thermocouple and a magnetic stirrer. In a typical reaction, 0.2 mmol catalyst and 20 mmol of styrene oxide were placed into the reactor before closing, introducing 2.0 MPa of CO_2_ and heating. After 18 h, the autoclave was cooled in an ice bath and slowly depressurized. Acetone was added to recover the reaction media and 1 mmol of 1,3,5-Trimethoxybenzene was added as internal standard for further ^1^H NMR analysis.

## 4. Conclusions

In conclusion, the mechanism of a CO_2_ cycloaddition into epoxides to form cyclic carbonates is an important reaction that is being studied intensively. In this paper and in order to contribute to this growing field, we performed an extensive catalyst study experimentally paired with theoretical results to propose and discuss the mechanistic aspect on the use of catalyst for such a reaction. We portrayed first a mechanism for the Lewis Based-catalysed reaction that is in accordance with the experimental results, the key factor being the stability of the first intermediate. We noted that this stability was dependent on the intramolecular interactions (H-bond) between the catalyst and the nucleophilic oxygen of the intermediate. We also showed that this non covalent interaction acts as a lock preventing the nucleophile from performing a dorsal attack for a ring closure.

In the second part of this study we proposed a reaction pathway for the Lewis acid-catalyzed mechanism, we found that it has the highest activation energy among all of the catalytic systems. This agrees well with the experimental results, a high E_A_ implies that competitive reactions might take place, explaining the poor yield. Lastly, we presented theoretical calculations for a plausible mechanism of the HIm/ZnI_2_-catalyzed reaction. The synergistic effect of imidazole and Zinc Iodide is reflected by the system performance both in experimental and computational results.

## Figures and Tables

**Table 1:** Variation of the catalyst for the CO_2_ cycloaddition into styrene oxide.

Entry	Lewis Base	ZnI_2_	SO Conversion (%)	SC Yield (%)
1	HIm	-	46	45
2	1M-Im	-	41	40
3	2M-HIm	-	54	53
4	Bz-HIm	-	31	31
5	2I-1M-Im	-	100	99
6	-	1.0 mol%	85	21
7	HIm	1.0 mol%	99	98

Reaction conditions: Styrene Oxide (20 mmol), Lewis Base (0.2 mmol), Lewis Acid (0.2 mmol), 20 bars of CO2, 100 °C, 18 h.

**Table 2:** Activation energies for step 1 (E_A1_) and step 2 (E_A2_) of the reaction pathway.

Catalyst	E_A1_ (kcal/mol) ^a^	E_A2_ (kcal/mol) ^b^
HIm	30.33	28.09
1M-Im	28.46	29.75
2M-HIm	28.92	24.93
Bz-HIm	30.26	29.09
2I-1M-Im	30.45	16.76

^a^ E_A1_ = G_TS1_ − G_Reactants_; ^b^ E_A2_ = G_TS2_ − G_I1_.

## Data Availability

All data supporting reported results are in the Supplementary Materials.

## Supplementary Materials

The following supporting information can be downloaded at: https://www.mdpi.com/article/10.3390/ijms24032123/s1. The supplementary contains: 1. Regioselectivity; 2. Non-covalent Interactions; and 3. Structures.

## References

[1] Callendar G.S. (1938). The artificial production of carbon dioxide and its influence on temperature. Q. J. R. Meteorol. Soc..

[2] Callendar G.S. (1941). Infra-red absorption by carbon dioxide, with special reference to atmospheric radiation. Q. J. R. Meteorol. Soc..

[3] Solomon S., Plattner G.-K., Knutti R., Friedlingstein P. (2009). Irreversible climate change due to carbon dioxide emissions. Proc. Natl. Acad. Sci. USA.

[4] Marchetti C. (1977). On geoengineering and the CO2 problem. Clim. Change.

[5] Marchetti C. (1979). Developments in Atmospheric Science.

[6] Metz B. (2005). Intergovernmental Panel on Climate Change. (Eds.) IPCC Special Report on Carbon Dioxide Capture and Storage.

[7] Artz J., Müller T.E., Thenert K., Kleinekorte J., Meys R., Sternberg A., Bardow A., Leitner W. (2018). Sustainable Conversion of Carbon Dioxide: An Integrated Review of Catalysis and Life Cycle Assessment. Chem. Rev..

[8] Yu Z., Li Z., Zhang L., Zhu K., Wu H., Li H., Yang S. (2021). A substituent- and temperature-controllable NHC-derived zwitterionic catalyst enables CO2 upgrading for high-efficiency construction of formamides and benzimidazoles. Green Chem..

[9] Zhao W., Chi X., Li H., He J., Long J., Xua Y., Yang S. (2019). Eco-friendly acetylcholine-carboxylate bio-ionic liquids for controllable N-methylation and N-formylation using ambient CO_2_ at low temperatures. Green Chem..

[10] Schäffner B., Schäffner F., Verevkin S., Börner A. (2010). Organic Carbonates as Solvents in Synthesis and Catalysis. Chem. Rev..

[11] Grignard B., Gennen S., Jérôme C., Kleij A.W., Detrembleur C. (2019). Advances in the use of CO_2_ as a renewable feedstock for the synthesis of polymers. Chem. Soc. Rev..

[12] Ke J., Li X., Wang F., Kang M., Feng Y., Zhao Y., Wang J. (2016). The hybrid polyhydroxyurethane materials synthesized by a prepolymerization method from CO_2_-sourced monomer and epoxy. J. CO2 Util..

[13] Bobbink F.D., van Muyden A.P., Dyson P.J. (2019). En route to CO_2_-containing renewable materials: Catalytic synthesis of polycarbonates and non-isocyanate polyhydroxyurethanes derived from cyclic carbonates. Chem. Commun..

[14] Pronoitis C., Hakkarainen M., Odelius K. (2022). Structurally Diverse and Recyclable Isocyanate-Free Polyurethane Networks from CO 2-Derived Cyclic Carbonates. ACS Sustain. Chem. Eng..

[15] Yang L.-C., Rong Z.-Q., Wang Y.-N., Tan Z.Y., Wang M., Zhao Y. (2017). Construction of Nine-Membered Heterocycles through Palladium-Catalyzed Formal [5+4] Cycloaddition. Angew. Chem. Int. Ed..

[16] Benin V., Gardelle B., Morgan A.B. (2014). Heat release of polyurethanes containing potential flame retardants based on boron and phosphorus chemistries. Polym. Degrad. Stab..

[17] Eisele A., Kyriakos K., Bhandary R., Schönhoff M., Papadakis C.M., Rieger B. (2015). Structure and ionic conductivity of liquid crystals having propylene carbonate units. J. Mater. Chem. A.

[18] Shi F., Zhang Q., Ma Y., He Y., Deng Y. (2005). From CO oxidation to CO2 activation: An unexpected catalytic activity of polymer-supported nanogold. J. Am. Chem. Soc..

[19] Rachuri Y., Kurisingal J.F., Chitumalla R.K., Vuppala S., Gu Y., Jang J., Choe Y., Suresh E., Park D.-W. (2019). Adenine-Based Zn(II)/Cd(II) Metal–Organic Frameworks as Efficient Heterogeneous Catalysts for Facile CO_2_ Fixation into Cyclic Carbonates: A DFT-Supported Study of the Reaction Mechanism. Inorg. Chem..

[20] Roshan K.R., Palissery R.A., Kathalikkattil A.C., Babu R., Mathai G., Lee H.-S., Park D.-W. (2016). A computational study of the mechanistic insights into base catalysed synthesis of cyclic carbonates from CO2: Bicarbonate anion as an active species. Catal. Sci. Technol..

[21] Kurisingal J.F., Li Y., Sagynbayeva Y., Chitumalla R.K., Vuppala S., Rachuri Y., Gu Y., Jang J., Park D.-W. (2020). Porous aluminum-based DUT metal-organic frameworks for the transformation of CO_2_ into cyclic carbonates: A computationally supported study. Catal. Today.

[22] Sun J., Wang J., Cheng W., Zhang J., Li X., Zhang S., She Y. (2012). Chitosan functionalized ionic liquid as a recyclable biopolymer-supported catalyst for cycloaddition of CO_2_. Green Chem..

[23] Xu K., Moeljadi A.M.P., Mai B.K., Hirao H. (2018). How Does CO_2_ React with Styrene Oxide in Co-MOF-74 and Mg-MOF-74? Catalytic Mechanisms Proposed by QM/MM Calculations. J. Phys. Chem. C.

[24] Rehman A., Eze V.C., Resul M.G., Harvey A. (2019). A kinetic study of Zn halide/TBAB-catalysed fixation of CO_2_ with styrene oxide in propylene carbonate. Green Process. Synth..

[25] Liu M., Liu B., Zhong S., Shi L., Liang L., Sun J. (2015). Kinetics and Mechanistic Insight into Efficient Fixation of CO_2_ to Epoxides over N-Heterocyclic Compound/ZnBr2 Catalysts. Ind. Eng. Chem. Res..

[26] Castro-Gmez F., Salassa G., Kleij A.W., Bo C. (2013). A DFT Study on the Mechanism of the Cycloaddition Reaction of CO_2_ to Epoxides Catalyzed by Zn(Salphen) Complexes. Chem. Eur. J..

[27] Lamine W., Boughdiri S., Christ L., Merzoud L., Morell C., Chermette H. (2020). Relaxation of Kohn–Sham orbitals of organometallic complexes during the approach of a nucleophilic reactant (or an electron approach): The case of [sal(ph)en]2 Zn complexes. Theor. Chem. Acc..

[28] Lamine W., Boughdiri S., Christ L., Morell C., Chermette H. (2016). Ill-advised self-interaction contribution in modelling anionic attack along a reaction path. Mol. Phys..

[29] Johnson E.R., Keinan S., Mori-Sánchez P., Contreras-García J., Cohen A.J., Yang W. (2010). Revealing Noncovalent Interactions. J. Am. Chem. Soc..

[30] Lu T., Chen Q. (2022). Independent gradient model based on Hirshfeld partition: A new method for visual study of interactions in chemical systems. J. Comput. Chem..

[31] Dudev T., Lim C. (2000). Tetrahedral vs Octahedral Zinc Complexes with Ligands of Biological Interest:  A DFT/CDM Study. J. Am. Chem. Soc..

[32] Congreve A., Kataky R., Knell M., Parker D., Puschmann H., Senanayake K., Wylie L. (2003). Examination of cobalt, nickel, copper and zinc(II) complex geometry and binding affinity in aqueous media using simple pyridylsulfonamide ligands. New J. Chem..

[33] Ataie N.J., Hoang Q.Q., Zahniser M.P.D., Tu Y., Milne A., Petsko G.A., Ringe D. (2008). Zinc Coordination Geometry and Ligand Binding Affinity: The Structural and Kinetic Analysis of the Second-Shell Serine 228 Residue and the Methionine 180 Residue of the Aminopeptidase from Vibrio proteolyticus. Biochemistry.

[34] Roy S., Sarkar B.N., Bhar K., Satapathi S., Mitra P., Ghosh B.K. (2013). Syntheses, structures and luminescence behaviors of zinc(II) complexes containing a tetradentate Schiff base: Variation in nuclearity and geometry with the change of halide/pseudohalide/carboxylate and counter anion. J. Mol. Struct..

[35] Gasque L., López-Rosales A., Bernès S., Mendoza-Díaz G. (2017). Bioinspired Co(II) and Zn(II) complexes with an imidazole derived tripodal ligand. Structural models for astacins and MnSOD. Polyhedron.

[36] Lamine W., Boughdiri S., Jeanneau E., Sanglar C., Morell C., Christ L., Chermette H. (2018). Unexpected Structure of a Helical N4-Schiff-Base Zn(II) Complex and Its Demetallation: Experimental and Theoretical Studies. ChemPhysChem.

[37] Lamine W., Boughdiri S., Christ L., Morell C., Chermette H. (2019). Coordination Chemistry of Zn2+ With Sal(ph)en Ligands: Tetrahedral Coordination or Penta-Coordination? A DFT Analysis. J. Comput. Chem..

[38] Paşaoğlu H., Güven S., Heren Z., Büyükgüngör O. (2006). Synthesis, spectroscopic and structural investigation of ZnI2(nicotinamide)2, ZnI2(isonicotinamide)2 and [Zn(H2O)2(picolinamide)2]I2. J. Mol. Struct..

[39] Bowmaker G.A., Effendy, Fariati, Rahajoe S.I., Skelton B.W., White A.H. (2011). Structural and Infrared Spectroscopic Studies of Some Adducts of Divalent Metal Dihalides (MX2, M = Zn, Cd; X = CI, Br, I) with Variously Hindered Monodentate Nitrogen (Pyridine) Base Ligands (L = Pyridine, 2-Methylpyridine, and Quinoline) of 1:2 Stoichiometry. Z. Anorg. Allg. Chem..

[40] Sun L., Zhang W.X., Ma J., Gao Y.L., Xu N., Pan C.Y., Lu T.Q., Hu X.Y., Jin F. (2017). Crystal structures and enhanced luminescence of Zn(II) and Cd(II) complexes containing conjugated organic ligands. Russ. J. Coord. Chem..

[41] Wang H., Cai F., Feng D., Zhou L., Li D., Wei Y., Feng Z., Zhang J., He J., Wu Y. (2019). Synthesis, crystal structure, photophysical property and bioimaging application of a series of Zn(II) terpyridine complexes. J. Mol. Struct..

[42] Chai J.-D., Head-Gordon M. (2008). Long-range corrected hybrid density functionals with damped atom–atom dispersion corrections. Phys. Chem. Chem. Phys..

[43] Hay P.J., Wadt W.R. (1985). Ab initio effective core potentials for molecular calculations. Potentials for K to Au including the outermost core orbitals. J. Chem. Phys..

[44] Hay P.J., Wadt W.R. (1985). Ab initio effective core potentials for molecular calculations. Potentials for the transition metal atoms Sc to Hg. J. Chem. Phys..

[45] Wadt W.R., Hay P.J. (1985). Ab initio effective core potentials for molecular calculations. Potentials for main group elements Na to Bi. J. Chem. Phys..

[46] Frisch M.J., Trucks G.W., Schlegel H.B., Scuseria G.E., Robb M.A., Cheeseman J.R., Scalmani G., Barone V., Mennucci B., Petersson G.A. (2010). Gaussian 09, Revision B.01.

[47] Frisch M.J., Trucks G.W., Schlegel H.B., Scuseria G.E., Robb M.A., Cheeseman J.R., Scalmani G., Barone V., Petersson G.A., Nakatsuji H. (2016). Gaussian 16, Revision B.01.

[48] Lu T., Chen F. (2012). Multiwfn: A multifunctional wavefunction analyzer. J. Comput. Chem..

