# Supplementary material for: Insight into the Varying Reactivity of Different Catalysts for CO2 Cycloaddition into Styrene Oxide: An Experimental and DFT Study
Source: Int J Mol Sci. 2023 Jan 20;24(3):2123. doi: 10.3390/ijms24032123 (PMC9916580; doi:10.3390/ijms24032123)
Supplement: Supplementary file 1 [file ijms-24-02123-s001.zip › ijms-2114168-supplementary.pdf]

Supporting Information for:

Insight into the Varying Reactivity of Different Catalysts for CO_2 Cycloaddition into Styrene Oxide: An Experimental and DFT Study

Angelo Pio Sebaaly [1,2], Hugo Dias [2], Lorraine Christ [2,*], Lynda Merzoud [1], Henry Chermette [1], Guillaume Hoffmann [1] and Christophe Morell [1,*]

1 Institut des Sciences Analytiques UMR CNRS 5280, Université Claude Bernard Lyon1, Université de Lyon, 5 Rue La Doua, 69622 Villeurbanne, France

2 Institut de Recherches sur la Catalyse et l'Environnement de Lyon, IRCELYON, UMR CNRS 5256, Université Claude Bernard Lyon1, Université de Lyon, 2 Av. Albert Einstein, 69626 Villeurbanne, France

* Correspondence: lorraine.christ@univ-lyon1.fr (L.C.); christophe.morell@univ-lyon1.fr (C.M.)

Table of Contents

1. Regioselectivity 2

2. Non-covalent Interactions 3

3. Structures 4

1. Regioselectivity

1-methylimidazole

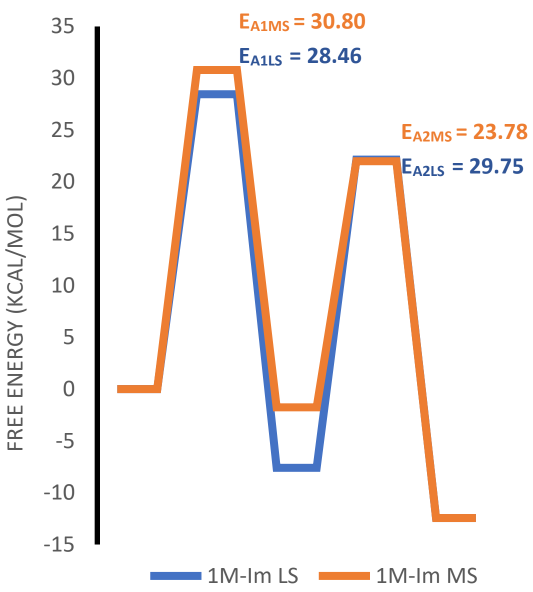

Figure S1. Free energy diagrams of 1M-Im catalyzed CO_2 cycloaddition, proceeding from the attack of the LB on the more substituted side of the epoxide in orange (1M-Im MS). In addition to that proceeding from the attack from the less substituted side in blue (1M-Im LS).

2-methylimidazole

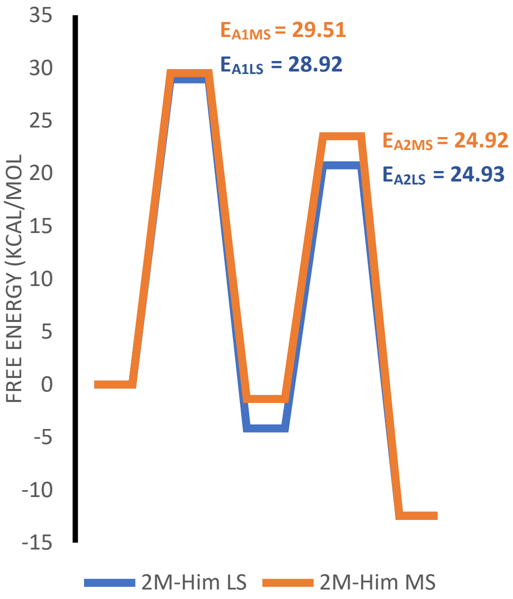

Figure S2. Free energy diagrams of 2M-HIm catalyzed CO_2 cycloaddition, proceeding from the attack of the LB on the more substituted side of the epoxide in orange (2M-HIm MS). In addition to that proceeding from the attack from the less substituted side in blue (2M-HIm LS).

2-iodo-1-methylimidazole

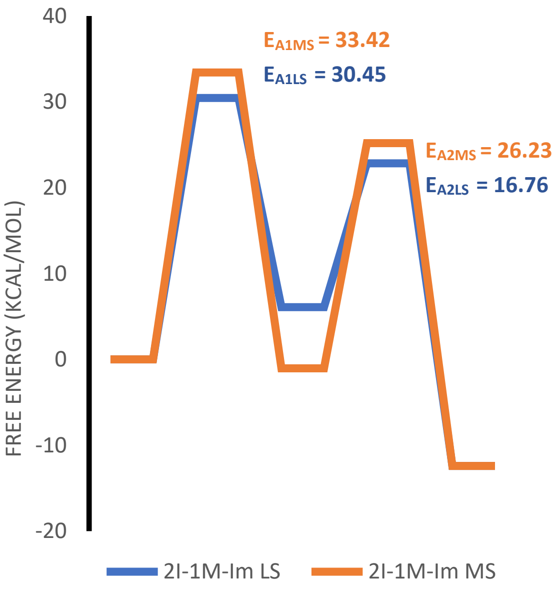

Figure S3. Free energy diagrams of 2I-1M-Im catalyzed CO_2 cycloaddition, proceeding from the attack of the LB on the more substituted side of the epoxide in orange (2I-1M-Im MS). In addition to that proceeding from the attack from the less substituted side in blue (2I-1M-Im LS).

Benzimidazole

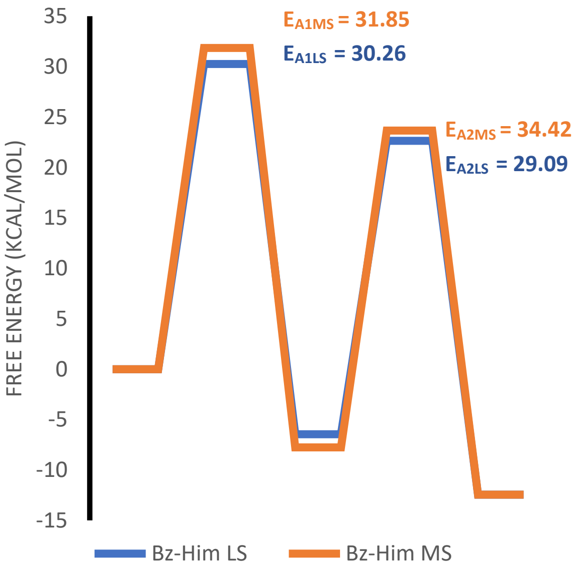

Figure S4. Free energy diagrams of Bz-HIm catalyzed CO_2 cycloaddition, proceeding from the attack of the LB on the more substituted side of the epoxide in orange (Bz-HIm MS). In addition to that proceeding from the attack from the less substituted side in blue (Bz-HIm LS).

2. Non-Covalent Interactions

Intermediate 1 1M-Im

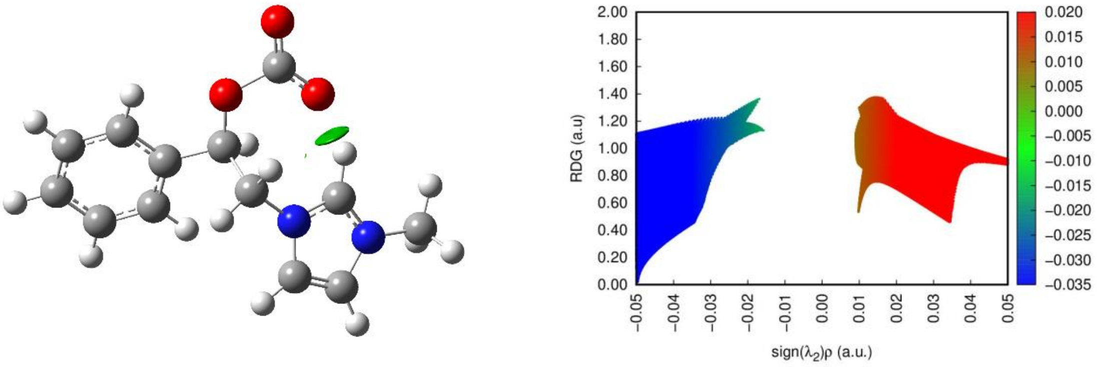

Figure S5. Visual representation of the NCI as a green surface (left). The Reduced Density Gradient (RDG) scatter graph representing the NCI of I1 1M-Im shown on the left

Intermediate 1 Bz-HIm

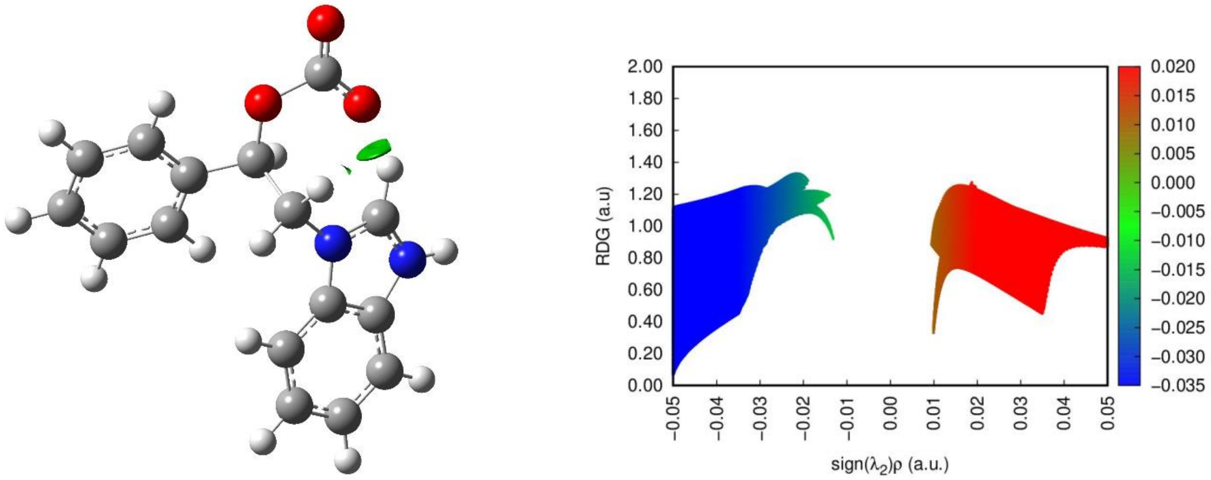

Figure S6. Visual representation of the NCI as a green surface (left). The Reduced Density Gradient (RDG) scatter graph representing the NCI of I1 Bz-HIm shown on the left

3. Structures

Imidazole

C	1.02456800	-0.50622300	-0.00005600
C	-1.14060500	-0.32655200	-0.00001200
C	-0.65435300	0.96800900	0.00009800
N	0.73074200	0.84230000	0.00005700
H	1.39658300	1.59721200	0.00011100
H	2.03010100	-0.89228000	-0.00010300
H	-2.16571500	-0.65619300	-0.00002800
H	-1.15314700	1.92156800	0.00019100
N	-0.08581100	-1.23968800	-0.00010700

1-methylimidazole

C	0.19401100	1.10190200	0.00079400
C	1.51483000	-0.61831900	-0.00009000
C	0.22559200	-1.12167100	0.00047900
N	-0.61546000	-0.01506800	-0.00009100
H	-0.19859800	2.10600100	0.00093500
H	2.44596200	-1.15970200	-0.00042100
H	-0.15005700	-2.13109900	0.00068600
N	1.48564400	0.77458100	-0.00063900
C	-2.07377800	-0.03495300	-0.00033000
H	-2.45270300	-0.54366700	-0.89081000
H	-2.45297600	-0.54125700	0.89140500
H	-2.44684900	0.99137300	-0.00180800

2-methylimidazole

C	-0.63277400	-0.07047400	0.00003200
C	1.44827800	-0.73154400	0.00001400
C	1.49555400	0.64838800	-0.00022700
N	0.16476600	1.05989900	0.00000500
H	-0.16160700	2.01232500	0.00001100
H	2.26741400	-1.43061600	0.00003500
H	2.31949100	1.34064700	-0.00038900
N	0.12250200	-1.16948800	0.00004900
C	-2.12772500	-0.02535500	0.00011000
H	-2.51757900	0.48438700	0.88815400
H	-2.51767100	0.48440200	-0.88788500
H	-2.50092200	-1.05011300	0.00012000

Benzimidazole

C	-0.92804200	1.44061700	0.00003500
C	0.25866700	0.69167800	0.00001500
C	0.26079600	-0.72813900	-0.00001600
C	-0.95319900	-1.43523900	-0.00002800
C	-2.13908100	-0.69817400	-0.00000600
C	-2.12533100	0.72063100	0.00002500
H	-0.92595800	2.52516100	0.00005700
H	-0.95370800	-2.51890400	-0.00005800
H	-3.09260000	-1.21528600	-0.00001600

H	-3.06844600	1.25710700	0.00004000
N	1.58492500	-1.20866600	-0.00004700
N	1.60637300	1.05170200	0.00001200
H	1.98152700	1.98520500	0.00006100
C	2.34269300	-0.12560100	0.00000200
H	3.42107800	-0.12916700	-0.00000200

2-iodo-1-methylimidazole

C	-0.65839300	-0.36087600	-0.00031600
C	-2.61986700	-1.27417500	-0.00075300
C	-2.83028400	0.09210100	-0.00002600
N	-1.56915700	0.68010000	-0.00037800
H	-3.34597500	-2.06869800	-0.00107600
H	-3.73398400	0.67718200	0.00005300
N	-1.25376500	-1.54588100	0.00043200
I	1.41195500	-0.06425300	0.00018800
C	-1.28199200	2.11204800	-0.00030200
H	-0.71320900	2.39137600	0.89038300
H	-0.71177400	2.39118800	-0.89012500
H	-2.22501500	2.66024700	-0.00116900

Styrene Oxide

C	-2.58669700	0.02538400	0.76796800
H	-2.22608100	-0.66180400	1.52803800
H	-3.49246200	0.57392200	1.00635000
C	-1.60030800	0.59978800	-0.19725300
H	-1.84069900	1.56140400	-0.64363500
O	-2.51534500	-0.49119900	-0.62069500
C	-0.14570700	0.27249800	-0.11555500
C	0.29338800	-1.06249100	-0.07469900
C	0.79843900	1.31226600	-0.06247500
C	1.66019200	-1.35101300	0.03377800
H	-0.43749800	-1.86113100	-0.15355300
C	2.16593100	1.02319000	0.04863300
H	0.46740000	2.34651500	-0.10732000
C	2.60020400	-0.31012800	0.09997900
H	1.99304500	-2.38345300	0.05978400
H	2.88806100	1.83193000	0.09011200
H	3.65835500	-0.53475500	0.18352700

Carbon Dioxide

C	0.00000000	0.00000000	0.00000000
O	0.00000000	0.00000000	1.18670700
O	0.00000000	0.00000000	-1.18670700

TS1 HIm LS

C	0.75653700	0.87739800	-0.82744200
H	0.48803000	0.52392800	-1.81494600
H	1.28897400	1.81482900	-0.72846300
C	0.94217900	-0.13479300	0.23911700

H	1.08864800	0.29550000	1.23424800
O	2.17580900	-0.31792900	-0.49589300
C	3.76386500	0.68682600	0.30076000
O	3.21129800	1.50005700	1.01244300
O	4.68166700	0.12929700	-0.23213600
C	-2.27662900	1.11782100	-0.77283200
C	-1.31464800	2.89397000	0.08460800
C	-3.28815800	1.90198300	-0.26049200
H	-2.33439600	0.15599600	-1.25233000
H	-0.58092000	3.61092200	0.41037200
H	-4.35339100	1.75712200	-0.23130700
H	-3.12229600	3.79520200	0.73711300
N	-2.66373400	3.02311500	0.28017800
N	-1.05840900	1.75399900	-0.55350300
C	0.00695400	-1.31409800	0.24416100
C	-1.02987300	-1.39617900	1.18884000
C	0.14058900	-2.32149600	-0.72567500
C	-1.93962400	-2.46324800	1.15368300
H	-1.12461500	-0.62763300	1.95175000
C	-0.76750200	-3.38956300	-0.76166000
H	0.97266700	-2.26085000	-1.41964300
C	-1.81297400	-3.46089400	0.17380700
H	-2.73522200	-2.52230900	1.88971700
H	-0.65524700	-4.16929500	-1.50817600
H	-2.51171400	-4.29099100	0.14837500

TS1 HIm MS

C	0.60669800	1.11042900	1.31461800
H	0.08321200	1.00232500	2.26310700
H	1.67985700	0.96665200	1.41983700
C	-0.00089300	0.48345400	0.13152100
H	0.58768700	0.55091200	-0.77211300
O	0.24834900	2.35127700	0.63027500
C	1.58652000	2.97618000	-0.37074400
O	2.39749000	2.05117800	-0.52531400
O	1.30794300	4.12816000	-0.62909800
C	0.83952600	-2.87802400	0.32025200
C	2.35159000	-1.35880600	-0.13928000
C	2.02864300	-3.55516200	0.13243800
H	-0.13433000	-3.26989600	0.55761500
H	2.83292800	-0.41124300	-0.33748800
H	2.26646000	-4.60324000	0.17978300
H	3.95278500	-2.73473500	-0.35106700
N	2.97643700	-2.57809400	-0.15835100
N	1.05800600	-1.51588300	0.14929000
C	-1.41647600	0.11494900	0.00499900
C	-1.84949800	-0.45329000	-1.20983700
C	-2.34348500	0.30037200	1.04814700
C	-3.18058800	-0.84672100	-1.37585000
H	-1.13432900	-0.59404900	-2.01473500
C	-3.67886400	-0.08246100	0.87835000
H	-2.03641800	0.77022800	1.97556900
C	-4.09920000	-0.66080800	-0.32986800
H	-3.50489500	-1.28578400	-2.31277600
H	-4.39260300	0.07567500	1.67934100

H	-5.13555700	-0.95459400	-0.45902000

Intermediate 1 HIm LS

C	-0.61842300	-0.10071200	-1.04676200
H	-0.02639000	-0.77038100	-1.67297100
H	-1.02003600	0.70546100	-1.66225700
C	0.25111600	0.53664100	0.09175400
H	-0.25755100	0.34970400	1.04783400
O	0.38138100	1.94198700	-0.12743100
C	-0.80768300	2.78279800	0.15781900
O	-1.84132100	2.10550800	0.52087100
O	-0.61531700	3.99131100	-0.01534000
C	-1.96721800	-2.25904600	-0.68295900
C	-2.73447000	-0.36554600	0.23688000
C	-3.12183000	-2.56910200	-0.01308600
H	-1.29895100	-2.88246200	-1.25000500
H	-2.70553600	0.70895200	0.51002200
H	-3.63431900	-3.50646100	0.10418100
H	-4.40644700	-1.26106800	1.11168200
N	-3.57768800	-1.37464000	0.54880100
N	-1.75103700	-0.89113200	-0.51684000
C	1.63576700	-0.08972300	0.12871300
C	1.77249500	-1.43102000	0.53127600
C	2.77350500	0.63756500	-0.24925100
C	3.03194000	-2.04557200	0.54534300
H	0.89753600	-1.99304100	0.85498000
C	4.03494800	0.02187000	-0.23540700
H	2.65204100	1.67613400	-0.53213200
C	4.16886500	-1.31838500	0.15716100
H	3.12969800	-3.07778900	0.86645700
H	4.91222700	0.59080200	-0.52551700
H	5.14677900	-1.78849100	0.17031500

Intermediate 1 HIm MS

C	-0.66586300	-1.43487100	0.01116500
H	-0.11340700	-2.36790100	-0.14296000
H	-0.76900800	-1.26364100	1.08989500
C	0.17253500	-0.32364400	-0.65287200
H	0.08649000	-0.46809000	-1.73477700
O	-1.94192800	-1.55883500	-0.62949300
C	-3.18317400	-1.36460700	0.17982700
O	-2.98981500	-0.66269500	1.24786800
O	-4.18973600	-1.86574000	-0.32862700
C	0.17079100	2.20637600	-0.91849400
C	-1.42640500	1.34208400	0.39912900
C	-0.58899200	3.24963600	-0.46690600
H	1.03591000	2.19429100	-1.55739600
H	-2.09545400	0.58538500	0.91818500
H	-0.51160800	4.30551400	-0.65237100
H	-2.31045900	3.18064000	0.82427000
N	-1.57125400	2.68592700	0.34933700
N	-0.36595700	1.03724300	-0.37253700
C	1.63045600	-0.39865300	-0.22967600

C	2.59610800	-0.87875200	-1.12733500
C	2.01605700	-0.04141100	1.07466900
C	3.93581300	-1.00192100	-0.72857900
H	2.30348600	-1.16394200	-2.13417300
C	3.35302700	-0.16070500	1.47335400
H	1.27470400	0.32646500	1.77922700
C	4.31664200	-0.64196600	0.57202800
H	4.67551800	-1.37648500	-1.42775400
H	3.64257700	0.11247600	2.48238600
H	5.35160000	-0.73813400	0.88202000

TS2 HIm LS

C	0.11384100	-1.38527800	-0.54622000
H	0.10189400	-0.96286000	-1.54095700
H	0.33252700	-2.44322000	-0.45628600
C	-0.49948200	-0.63461400	0.65981200
H	0.18035200	-0.72205100	1.51078700
O	-1.71324000	-1.28607700	1.10580200
C	-2.43314900	-1.96304200	0.04324300
O	-1.72309200	-1.97923900	-1.05566700
O	-3.54394100	-2.42293600	0.31127400
C	2.43755900	0.36646600	-0.64511000
C	2.87182300	-1.60494500	0.23538500
C	3.78216400	0.35150500	-0.34815400
H	1.83506900	1.14834500	-1.07395100
H	2.76886400	-2.60827000	0.61074400
H	4.54361800	1.09932900	-0.47898500
H	4.93432100	-1.23093000	0.53197300
N	4.03938100	-0.89894100	0.20792400
N	1.89316300	-0.85840000	-0.27352200
C	-0.72216500	0.83628600	0.34747700
C	-0.14413800	1.82151500	1.16180300
C	-1.50544400	1.22619800	-0.75560400
C	-0.33224200	3.18347600	0.87971900
H	0.44607900	1.52583500	2.02548400
C	-1.69541800	2.58475500	-1.03785000
H	-1.97028100	0.46609400	-1.37690100
C	-1.10709200	3.56789500	-0.22422500
H	0.11329900	3.93694800	1.52112300
H	-2.30791400	2.87724900	-1.88425900
H	-1.26098700	4.61964500	-0.44251200

TS2 HIm MS

C	-0.32387400	-1.83317400	-0.53981700
H	0.25817800	-1.66413300	-1.44825000
H	-0.03794800	-2.79614600	-0.09955700
C	-0.05744000	-0.72606000	0.49486000
H	0.14174300	-1.00402500	1.51996000
O	-1.69742900	-1.86443600	-0.96330300
C	-2.62377100	-1.60277300	0.13779400
O	-1.97439700	-1.20413500	1.19524300
O	-3.82358700	-1.77175700	-0.09082000
C	2.76685300	-1.55766100	0.41693300
C	2.46682700	0.48546700	-0.34653300

C	4.01119400	-1.07856000	0.07307000
H	2.49324700	-2.51287300	0.82989000
H	2.00828000	1.41224800	-0.64705500
H	4.98513900	-1.53112300	0.12995500
H	4.51439700	0.84224300	-0.74100400
N	3.80276800	0.21111200	-0.40732100
N	1.82365600	-0.57155600	0.14740000
C	-0.43807600	0.69391300	0.23280400
C	-0.36433300	1.62729400	1.28135500
C	-0.82655300	1.12267500	-1.04888100
C	-0.66626500	2.97451000	1.05485000
H	-0.09696100	1.28869100	2.27754600
C	-1.13079100	2.47250700	-1.27532600
H	-0.93318000	0.40444000	-1.85445000
C	-1.04575900	3.40245600	-0.22850900
H	-0.62245400	3.68507800	1.87329400
H	-1.45026700	2.79294200	-2.26117700
H	-1.28991600	4.44484900	-0.40426700

Product HIm

C	-0.69299600	-1.56806300	-0.50772500
H	-0.15912300	-1.10954100	-1.33584600
H	-0.19388700	-2.48452400	-0.19719600
C	-0.92515500	-0.60007600	0.67927400
H	-0.32713500	-0.88808500	1.53917700
O	-2.34415400	-0.86684000	1.02602400
C	-2.97606500	-1.55562700	0.00783700
O	-2.05042900	-1.90251400	-0.96472200
O	-4.16216000	-1.81701300	-0.03496100
C	2.61178100	0.04989200	-0.48471700
C	3.20120700	-1.76743500	0.56064000
C	3.94331600	-0.15114600	-0.78983800
H	1.96353200	0.85015800	-0.80151500
H	3.20365500	-2.65688200	1.16759800
H	4.62889300	0.41056400	-1.39974600
H	5.21972200	-1.74201100	-0.12479600
N	4.30933000	-1.31195100	-0.11732200
N	2.15699600	-0.96629500	0.36069400
C	-0.73458400	0.86365000	0.36275100
C	0.07030100	1.65855600	1.19112000
C	-1.35514500	1.44304000	-0.75939300
C	0.26073800	3.01829800	0.90381600
H	0.56072800	1.21053600	2.04958100
C	-1.16812000	2.80019200	-1.04784800
H	-1.98626800	0.83996500	-1.40729200
C	-0.35824500	3.59182500	-0.21650100
H	0.88651000	3.62471500	1.54990600
H	-1.65368900	3.24094600	-1.91195800
H	-0.21547100	4.64417700	-0.43872000

TS1 1M-Im LS

C	-0.53526800	-1.00645000	-0.90840100
H	-0.55693600	-0.53093100	-1.88057200
H	-0.46192600	-2.08462300	-0.84753700
C	-1.16756100	-0.29217000	0.22467700

H	-0.99383200	-0.76344400	1.19654900
O	-2.34475500	-0.79105600	-0.45546300
C	-3.08446300	-2.53029400	0.34282000
O	-2.13488000	-2.92742800	0.98460700
O	-4.18770700	-2.54432700	-0.12495100
C	2.16662700	0.42484300	-0.97012900
C	2.34444900	-1.59946200	-0.15381000
C	3.45997600	0.29232500	-0.50817800
H	1.68390600	1.27783700	-1.41511400
H	2.12336600	-2.60420600	0.16516300
H	4.28432400	0.98428500	-0.49840500
N	3.56200300	-0.99604200	0.00928800
N	1.48777300	-0.76628100	-0.74598300
C	-1.02628600	1.20557000	0.26693600
C	-0.12474600	1.81011800	1.15917200
C	-1.76784800	2.00719800	-0.61647700
C	0.05123300	3.20157900	1.15662700
H	0.43816400	1.19331000	1.85515200
C	-1.59405000	3.39886600	-0.61941500
H	-2.48662600	1.52315200	-1.26962900
C	-0.68095000	3.99954800	0.26282100
H	0.74662000	3.66176800	1.85156900
H	-2.17491200	4.01441800	-1.29885900
H	-0.55120200	5.07713000	0.26302800
C	4.75471000	-1.58989700	0.61273800
H	5.56271000	-1.64760000	-0.12003000
H	5.08148900	-0.99469100	1.46825300
H	4.52022400	-2.59830100	0.95746000

TS1 1M-Im MS

C	-0.34396500	1.31568300	1.29101900
H	-0.70466000	0.92089200	2.24013400
H	0.63027200	1.79215200	1.38321200
C	-0.52679000	0.47508700	0.09951900
H	-0.05866900	0.84812800	-0.80097800
O	-1.33876500	2.14663600	0.61721700
C	-0.57057000	3.45684700	-0.38544600
O	0.61021600	3.13140800	-0.54514900
O	-1.45523400	4.25221000	-0.61189900
C	1.74277300	-2.04952400	0.35847100
C	2.44461000	-0.02140000	-0.06689100
C	3.12011900	-2.11555600	0.26442300
H	1.03298400	-2.83520300	0.55344200
H	2.46683100	1.03910000	-0.27071700
H	3.80241600	-2.94245900	0.36387700
N	3.55448300	-0.82113200	-0.00639700
N	1.33943800	-0.73851300	0.15046300
C	-1.55355500	-0.57576700	-0.02452400
C	-1.54730200	-1.39121900	-1.17267800
C	-2.53252000	-0.78445300	0.96395300
C	-2.48889200	-2.41461600	-1.32175400
H	-0.79314000	-1.22788500	-1.93669300
C	-3.48230800	-1.80147300	0.80972500
H	-2.58212900	-0.12977800	1.82664900
C	-3.45966000	-2.62252300	-0.32831500

H	-2.47451800	-3.03999500	-2.20779700
H	-4.24415500	-1.94711000	1.56779300
H	-4.19816700	-3.40863800	-0.44562600
C	4.94118200	-0.39634700	-0.19368600
H	5.38295200	-0.90612000	-1.05298700
H	4.96538900	0.67954100	-0.37386300
H	5.52867700	-0.61558600	0.70084300

Intermediate 1 1M-Im LS

C	0.24242600	-0.02753400	-1.16278900
H	-0.32474900	0.67856000	-1.77197900
H	0.52924300	-0.88228500	-1.77708300
C	-0.60803000	-0.55476000	0.04398900
H	-0.02128200	-0.39981900	0.96016200
O	-0.88574300	-1.94538000	-0.12904300
C	0.23176100	-2.89160100	0.11280600
O	1.34453300	-2.31159000	0.40153800
O	-0.08606000	-4.07940400	-0.01782000
C	1.80881900	1.99961200	-0.93942400
C	2.44759400	0.06735200	-0.01250100
C	3.02637400	2.20487200	-0.34282100
H	1.17301000	2.67492800	-1.48417900
H	2.33756300	-0.99061800	0.29310300
H	3.63469900	3.08952100	-0.27935800
N	3.40525300	0.98740500	0.22866300
N	1.47199400	0.66617400	-0.72470500
C	-1.92103900	0.20345700	0.15356800
C	-1.90155800	1.56172200	0.52084900
C	-3.14652400	-0.41941600	-0.12318000
C	-3.09292200	2.29547800	0.59994700
H	-0.95645200	2.04454800	0.76528100
C	-4.33982400	0.31564000	-0.04418100
H	-3.14427900	-1.47158600	-0.38069200
C	-4.31824100	1.67220500	0.31311500
H	-3.06942200	3.34038900	0.89294700
H	-5.28527300	-0.17289600	-0.25633600
H	-5.24379800	2.23509700	0.37673200
C	4.63982800	0.73522900	0.97938800
H	5.50739300	0.90760400	0.33974400
H	4.68650000	1.39120700	1.85056200
H	4.64230000	-0.30223800	1.31339100

Intermediate 1 1M-Im MS

C	0.13738500	1.28162400	1.12861500
H	-0.59139100	1.25724100	1.94178700
H	1.12273900	1.01378300	1.52655700
C	-0.23452500	0.26636300	0.00351500
H	0.00070600	0.76830700	-0.93888200
O	0.14655200	2.62051500	0.62425500
C	1.28733100	2.95548200	-0.27383300
O	2.03521400	1.94168400	-0.55128600
O	1.32032600	4.14293500	-0.61726300
C	0.58340500	-2.10729000	0.70879800
C	1.99496900	-0.73636000	-0.34910500
C	1.80224700	-2.73127100	0.63910300

H	-0.34386300	-2.43318200	1.14430400
H	2.35468400	0.22431200	-0.74028500
H	2.11480300	-3.69531000	0.99938700
N	2.66884500	-1.85853100	-0.02415300
N	0.72219200	-0.87621100	0.07379000
C	-1.68788100	-0.18403600	-0.05034900
C	-2.07067300	-1.35919000	-0.72597700
C	-2.69123000	0.65992700	0.45761700
C	-3.42171900	-1.71071600	-0.84326700
H	-1.32009500	-1.99791300	-1.18296700
C	-4.04357800	0.31177100	0.33449400
H	-2.42682800	1.60597300	0.91648600
C	-4.41490500	-0.87900900	-0.30540600
H	-3.69773100	-2.62161200	-1.36414600
H	-4.80415600	0.97816500	0.72671400
H	-5.46202000	-1.14549700	-0.40056500
C	4.08767400	-2.09973100	-0.30610300
H	4.19468900	-2.91412200	-1.02522100
H	4.52358900	-1.19204000	-0.72344200
H	4.60986700	-2.35135700	0.61855400

TS2 1M-Im LS

C	-0.07009300	-1.31442900	-0.60913500
H	-0.18124300	-0.88463000	-1.59435500
H	0.31397100	-2.32588400	-0.54489100
C	-0.72712100	-0.67115200	0.63497900
H	-0.00401100	-0.67029500	1.45379800
O	-1.81667700	-1.49300300	1.12076700
C	-2.47050700	-2.26116000	0.08019900
O	-1.80986800	-2.16612900	-1.04603200
O	-3.49083000	-2.88141500	0.38300000
C	1.98043000	0.75996400	-0.78391400
C	2.72828300	-1.12136100	0.07185300
C	3.32378100	0.93509100	-0.53098300
H	1.25895700	1.44897600	-1.18803700
H	2.78555300	-2.12907200	0.44703100
H	3.96659400	1.78444000	-0.68389500
N	3.78531000	-0.26133100	0.01139300
N	1.63052100	-0.52755000	-0.40042200
C	-1.17077100	0.75578600	0.35731600
C	-0.69646000	1.80638600	1.15657500
C	-2.05657900	1.03961900	-0.69956400
C	-1.08899200	3.13019000	0.90481000
H	-0.02586000	1.59035200	1.98438900
C	-2.45058500	2.35972300	-0.95155600
H	-2.43975700	0.22657500	-1.30943900
C	-1.96599200	3.40976900	-0.15327400
H	-0.72141500	3.93414600	1.53426900
H	-3.14038700	2.56929900	-1.76236400
H	-2.27749500	4.43087400	-0.34792400
C	5.16016200	-0.54098600	0.43144400
H	5.83812400	-0.45979500	-0.42058100
H	5.46519300	0.16137700	1.20970600
H	5.21662600	-1.55446400	0.83112400

TS2 1M-Im MS

C	-0.82365100	-1.81359100	-0.55170800
H	-0.17639300	-1.69281100	-1.42260100
H	-0.67214300	-2.81133500	-0.12252100
C	-0.49440900	-0.76458600	0.52397600
H	-0.37700500	-1.08197400	1.55005700
O	-2.16782100	-1.68128600	-1.04710700
C	-3.11318000	-1.32794300	0.00846700
O	-2.47814500	-1.01720900	1.10461500
O	-4.31159600	-1.35574000	-0.28016300
C	2.22580000	-1.91200900	0.58551000
C	2.20466800	0.14852100	-0.17830500
C	3.53157600	-1.57353500	0.30364300
H	1.82700900	-2.83064000	0.97961700
H	1.87070900	1.12239900	-0.49562900
H	4.44616700	-2.13187700	0.40475500
N	3.50379900	-0.26804500	-0.17852400
N	1.41623000	-0.82718700	0.27740100
C	-0.68452000	0.69528400	0.27297100
C	-0.54847900	1.59838200	1.34138300
C	-0.95491200	1.18592200	-1.01674700
C	-0.67484500	2.97527500	1.12761500
H	-0.37034500	1.21571700	2.34150100
C	-1.08247000	2.56566000	-1.23087700
H	-1.10891800	0.49600100	-1.83936400
C	-0.93741400	3.46408800	-0.16325700
H	-0.58546700	3.66395100	1.96101900
H	-1.31195700	2.93609000	-2.22427800
H	-1.04822300	4.53049900	-0.32954200
C	4.66314200	0.51385300	-0.61330900
H	4.33996000	1.52338400	-0.87081400
H	5.39758300	0.57503600	0.19217100
H	5.12147700	0.05414400	-1.49142700

TS1 2M-HIm LS

C	-0.87672700	0.34088800	1.06201400
H	-0.49899600	-0.05514400	1.99628300
H	-1.59800800	1.14517000	1.08416000
C	-0.81234600	-0.52564800	-0.13440900
H	-1.02415400	-0.01194400	-1.07701300
O	-1.99686300	-1.04759400	0.51750700
C	-3.72599500	-0.21195000	-0.22914100
O	-3.29812000	0.79160500	-0.76220900
O	-4.54348400	-1.00330100	0.14165000
C	2.00908700	1.50368900	1.20352800
C	0.63222000	2.65075600	-0.08210300
C	2.79125000	2.38733400	0.49493200
H	2.30510300	0.75282500	1.91490000
H	3.85389000	2.55232400	0.48869700
H	2.15901500	3.83581000	-0.95861900
N	1.90920100	3.10400600	-0.31245800
N	0.67645100	1.68489000	0.84154800
C	0.34930800	-1.47621100	-0.24141200
C	1.47097000	-1.13663400	-1.01732000
C	0.33373900	-2.69223000	0.45968000

C	2.57554400	-1.99745200	-1.08099800
H	1.47959500	-0.20116100	-1.57195500
C	1.43718000	-3.55600000	0.39358000
H	-0.55340900	-2.94534800	1.03052600
C	2.56156400	-3.21005300	-0.37275800
H	3.43799600	-1.72989500	-1.68334800
H	1.41790400	-4.49852200	0.93130400
H	3.41311800	-3.88092500	-0.42530700
C	-0.58861100	3.18991400	-0.75783500
H	-1.04776800	3.98376900	-0.15992500
H	-0.34250300	3.60176100	-1.74083800
H	-1.33670400	2.40490100	-0.89463000

TS1 2M-HIm MS

C	0.89325700	-0.96194200	-1.31330700
H	0.38861800	-0.87313600	-2.27396200
H	1.92477300	-0.61533000	-1.35413200
C	0.15014600	-0.58194300	-0.10578900
H	0.73674100	-0.54870000	0.80212000
O	0.74114100	-2.29105300	-0.72322000
C	2.18384400	-2.80101300	0.27602500
O	2.81770700	-1.77165600	0.51968700
O	2.07026500	-3.99790700	0.41198300
C	-0.55511400	2.61797900	-0.27110500
C	1.58774800	2.27189400	0.10753000
C	0.02841900	3.86405000	-0.20918600
H	-1.58670600	2.35346300	-0.43306300
H	-0.38593700	4.85175000	-0.30864700
H	2.09463600	4.33549700	0.13580200
N	1.38157800	3.63135200	0.03069800
N	0.42209400	1.64533100	-0.07465400
C	-1.31788100	-0.58817600	0.01829400
C	-1.88250800	-0.34565300	1.28497300
C	-2.16690900	-0.80005400	-1.08336300
C	-3.27100700	-0.30697000	1.45031000
H	-1.22860700	-0.18098200	2.13607900
C	-3.55674900	-0.76662000	-0.91898600
H	-1.75008200	-1.01373400	-2.06102200
C	-4.11244200	-0.51734200	0.34600200
H	-3.69621200	-0.12227200	2.43063900
H	-4.20558500	-0.94254200	-1.77009400
H	-5.18984800	-0.49516200	0.47167900
C	2.92300100	1.64342700	0.35389800
H	3.59601100	1.80241300	-0.49565900
H	3.39676400	2.06560200	1.24614500
H	2.82398800	0.56547000	0.50243100

Intermediate 1 2M-HIm LS

C	-0.68410700	-0.16847700	1.03486300
H	-0.06200400	-0.08132300	1.92793900
H	-1.55745800	-0.78459100	1.24794000
C	0.10698800	-0.83976700	-0.13001800
H	-0.43349400	-0.62073000	-1.06106800
O	0.12630500	-2.24433400	0.11845400

C	-1.16457000	-2.96762200	-0.18942300
O	-2.09631600	-2.18227100	-0.58384900
O	-1.09060800	-4.18559000	0.01316100
C	-0.41383700	2.35865200	0.82871600
C	-2.28715200	1.44494300	-0.01417300
C	-1.13176100	3.37106100	0.25557200
H	0.54562600	2.36315500	1.31447000
H	-0.91953100	4.42067000	0.16820700
H	-3.03081300	3.26066200	-0.74605800
N	-2.28795700	2.78165000	-0.26047900
N	-1.14525200	1.18179000	0.65841900
C	1.52442600	-0.30947200	-0.19905300
C	1.83659900	0.78852800	-1.01772600
C	2.53235500	-0.88614700	0.59369300
C	3.13440900	1.32421900	-1.02974500
H	1.07295200	1.21195900	-1.66655300
C	3.82832800	-0.35367600	0.58202700
H	2.29156900	-1.76617100	1.17998400
C	4.13230500	0.75726400	-0.22339400
H	3.36852600	2.16477400	-1.67532900
H	4.60416600	-0.81057200	1.18770600
H	5.13886900	1.16255500	-0.23581500
C	-3.35090400	0.47606600	-0.38907000
H	-4.04895400	0.33974800	0.44500600
H	-3.91005900	0.83885900	-1.25523300
H	-2.92046000	-0.51895200	-0.61252200

Intermediate 1 2M-HIm MS

C	-0.53046600	1.01085400	-1.25579800
H	0.23481500	1.11266900	-2.03106600
H	-1.42315100	0.52729500	-1.66697300
C	0.00375100	0.14364900	-0.07094700
H	-0.42819000	0.58002100	0.83124500
O	-0.83505400	2.33367700	-0.80843400
C	-2.07571400	2.43569700	0.02385600
O	-2.61501700	1.29159100	0.25689100
O	-2.35199500	3.59549800	0.35871900
C	-0.30652400	-2.09572300	-1.23556400
C	-1.54875600	-1.72065900	0.59825700
C	-1.13917600	-3.16789100	-1.09022100
H	0.44014500	-1.87915600	-1.97855400
H	-1.25463000	-4.05530500	-1.68502700
H	-2.62133300	-3.51011400	0.42903100
N	-1.89722900	-2.91598400	0.05585800
N	-0.56971400	-1.21605900	-0.18184000
C	1.52065200	0.12594100	0.05989500
C	2.25030800	-1.02024900	0.41573900
C	2.20082000	1.34699800	-0.11489300
C	3.64402700	-0.95573800	0.57189900
H	1.74647500	-1.96838800	0.58130000
C	3.58975000	1.40975200	0.04559800
H	1.63627600	2.24311800	-0.35732100
C	4.31828600	0.25807800	0.38507000
H	4.19693200	-1.84904900	0.84365900
H	4.10126400	2.35679000	-0.08821900

H	5.39468200	0.30969400	0.50927700
C	-2.16119600	-1.09468100	1.79593700
H	-2.93398200	-1.74162300	2.21706600
H	-1.40551700	-0.89966400	2.56291200
H	-2.59323100	-0.12428000	1.48415900

TS2 2M-HIm LS

C	-0.22845500	1.14905500	-0.71900200
H	-0.04626800	0.67600700	-1.67346300
H	-0.75474300	2.09571400	-0.72384600
C	0.47517100	0.66418300	0.57088300
H	-0.25881800	0.60873300	1.37832100
O	1.43638700	1.64659800	1.02775000
C	1.99468700	2.44708200	-0.04194500
O	1.37596400	2.20202500	-1.17044000
O	2.91024300	3.22146000	0.23847300
C	-2.00425400	-1.16373300	-0.92971000
C	-2.93849800	0.54737800	0.11883900
C	-3.28303400	-1.55292000	-0.60893900
H	-1.21965000	-1.71157000	-1.42233600
H	-3.80835800	-2.47536100	-0.77938500
H	-4.79488700	-0.42586700	0.41832900
N	-3.85700700	-0.46596000	0.05069700
N	-1.81285900	0.13701900	-0.47330800
C	1.10110600	-0.70646300	0.37455100
C	0.70532600	-1.78225200	1.18246600
C	2.07440100	-0.91346200	-0.62124400
C	1.26439400	-3.05671300	1.00056200
H	-0.03582600	-1.62448000	1.96200000
C	2.63493900	-2.18379700	-0.80299900
H	2.39175500	-0.08036000	-1.24151400
C	2.23037600	-3.26024200	0.00468000
H	0.95532200	-3.88049600	1.63577800
H	3.39084500	-2.33354100	-1.56675500
H	2.67046300	-4.24220400	-0.13543200
C	-3.17421500	1.88451900	0.74286300
H	-3.31964700	2.65284600	-0.02242900
H	-4.05805400	1.86847100	1.38521900
H	-2.31549400	2.17821700	1.35149400

TS2 2M-HIm MS

C	-0.55028400	-1.74986800	-0.67201400
H	0.00095100	-1.46047100	-1.57008100
H	-0.23357400	-2.75303100	-0.36355800
C	-0.25138200	-0.76725700	0.47342600
H	-0.07786200	-1.15014900	1.46903100
O	-1.93626500	-1.76059400	-1.05820900
C	-2.84120200	-1.57415800	0.07549500
O	-2.17599400	-1.22362300	1.13888600
O	-4.04340500	-1.74546600	-0.13911600
C	2.42969400	-1.70170900	0.84354200
C	2.46342100	0.02980600	-0.53628900
C	3.74716400	-1.49432400	0.51675200
H	1.99326600	-2.44588800	1.48777800

H	4.64356600	-2.00858300	0.81345700
H	4.56468600	0.00949700	-0.77709800
N	3.75020900	-0.40414900	-0.35017700
N	1.65074300	-0.75179100	0.18687800
C	-0.54501500	0.69368500	0.34816900
C	-0.12611400	1.56355700	1.36903400
C	-1.20418600	1.21615600	-0.77762200
C	-0.33785300	2.94265400	1.25907000
H	0.36286600	1.15731400	2.24979900
C	-1.42105900	2.59572800	-0.88616100
H	-1.57515900	0.54659100	-1.54696200
C	-0.98132600	3.46445800	0.12430100
H	-0.01684500	3.60643500	2.05490900
H	-1.94393600	2.99020300	-1.75073500
H	-1.15488000	4.53205300	0.03907700
C	2.09804600	1.18837700	-1.40563200
H	2.97467600	1.54931400	-1.95051700
H	1.33106600	0.90433500	-2.12870900
H	1.69040900	2.00880100	-0.80820300

TS1 Bz-HIm LS

C	-1.34808800	-0.87738700	-0.79735100
H	-1.02861800	-0.45944200	-1.74344500
H	-1.78854200	-1.86625000	-0.78248400
C	-1.73298000	0.06518800	0.27950700
H	-1.91853800	-0.41796700	1.24335000
O	-2.91056400	0.15192700	-0.55790300
C	-4.43574800	-1.05266700	0.05276600
O	-3.85824800	-1.84151100	0.77297300
O	-5.36424600	-0.56828200	-0.53080100
C	1.73544900	-0.93096900	-0.47901700
C	0.74185000	-2.72773400	0.28707500
C	2.73373500	-1.71578800	0.14547700
H	-0.00669700	-3.45994500	0.54290700
H	2.47952300	-3.62170500	1.12378700
N	2.06838300	-2.85113800	0.62266100
N	0.50528900	-1.60502100	-0.37353500
C	-0.92548100	1.32854100	0.40198700
C	0.02928900	1.46504900	1.42293200
C	-1.08057800	2.35545700	-0.54346200
C	0.84429300	2.60403900	1.48417900
H	0.14047000	0.67842800	2.16486000
C	-0.26682300	3.49632600	-0.48306500
H	-1.85368200	2.24842700	-1.29760800
C	0.70283900	3.62008600	0.52591100
H	1.58353500	2.69976500	2.27276300
H	-0.39481400	4.29053400	-1.21168900
H	1.33034900	4.50432700	0.57364900
C	2.05250400	0.30882300	-1.05703200
H	1.28807700	0.93281000	-1.50445100
C	4.07402800	-1.30651300	0.20997600
H	4.83884200	-1.90720600	0.68872800
C	3.38279700	0.72140000	-0.99561200
H	3.66149300	1.67856400	-1.42100100
C	4.37891200	-0.07543000	-0.37419500

H	5.40138500	0.28516600	-0.34786400

TS1 Bz-HIm MS

C	-1.90959900	-0.70430600	-1.31102500
H	-1.69177700	-0.21328500	-2.25841000
H	-1.81073700	-1.78679700	-1.37670600
C	-1.35809800	-0.10173000	-0.08832200
H	-1.51533300	-0.68247000	0.81069700
O	-3.18100200	-0.26991700	-0.74160200
C	-3.97926700	-1.57325100	0.22780700
O	-3.11273800	-2.42199300	0.46733600
O	-5.12873800	-1.21739800	0.37167300
C	1.97800600	-0.58868500	-0.09235500
C	0.68292100	-2.32280000	0.23630200
C	2.83089800	-1.70326700	0.10389100
H	-0.19032100	-2.94470000	0.36908900
H	2.24497400	-3.74458400	0.48459200
N	1.97485400	-2.79036100	0.31062800
N	0.64470600	-1.02045600	-0.00335800
C	-0.98756500	1.31548300	0.05963900
C	-0.57586600	1.76695400	1.32812200
C	-0.99035100	2.21284300	-1.02362200
C	-0.15106700	3.08692800	1.50988600
H	-0.57595800	1.07554500	2.16526900
C	-0.57217100	3.53655200	-0.84211000
H	-1.33559500	1.89075900	-1.99931800
C	-0.14515600	3.97487500	0.42190100
H	0.16840400	3.42438700	2.48948600
H	-0.58519400	4.22647100	-1.67869000
H	0.17807700	5.00120000	0.55997900
C	2.51013300	0.69019500	-0.32817700
H	1.86192900	1.54587400	-0.47721300
C	3.90007700	0.80974600	-0.36015000
H	4.34799500	1.78092400	-0.53871200
C	4.22850500	-1.58834900	0.07144400
H	4.88145300	-2.44073100	0.22029800
C	4.74591800	-0.31256900	-0.16375900
H	5.82107200	-0.17412600	-0.19735100

Intermediate 1 Bz-HIm LS

C	-0.25858700	-0.59086300	-0.98013600
H	-0.21103600	0.32440200	-1.57164000
H	-0.49115400	-1.42613100	-1.64150700
C	-1.37163100	-0.50426000	0.11973800
H	-0.91022200	-0.76980200	1.08132300
O	-2.42820100	-1.41676300	-0.18438100
C	-2.14746600	-2.85991100	0.00797800
O	-0.93469900	-3.10488300	0.36827500
O	-3.11605600	-3.59168100	-0.22469400
C	2.16246700	0.08764100	-0.38528000
C	1.42889100	-1.93516000	0.24673100
C	3.21069200	-0.55804700	0.30081100
H	0.67075000	-2.73033100	0.39531700
H	3.19336500	-2.52711700	1.19468500
N	2.70536900	-1.81516900	0.67452600

N	1.08116000	-0.81237100	-0.40216300
C	-1.93976200	0.90305100	0.20324500
C	-1.12271200	1.94639800	0.67680300
C	-3.25152500	1.18369200	-0.20484000
C	-1.60651700	3.26049400	0.73172600
H	-0.11273000	1.73248100	1.02334200
C	-3.73579000	2.50024500	-0.15037600
H	-3.87291600	0.36371300	-0.54391900
C	-2.91733000	3.54131500	0.31293000
H	-0.97269300	4.05712100	1.10843100
H	-4.75295700	2.71064400	-0.46442600
H	-3.29777500	4.55663200	0.35730800
C	2.30639200	1.38635800	-0.88957600
H	1.49890200	1.89463300	-1.40265400
C	4.45078500	0.05908800	0.50287100
H	5.25918000	-0.43461200	1.02865200
C	3.54212800	2.00541100	-0.68851500
H	3.69746500	3.01119000	-1.06074400
C	4.59789000	1.35218800	-0.00596000
H	5.54022100	1.87072900	0.12678100

Intermediate 1 Bz-HIm MS

C	-1.62024100	1.60200400	0.25378100
H	-1.47751400	2.62436400	0.61971300
H	-1.78345200	1.63931000	-0.83061100
C	-0.32101200	0.84019900	0.59168100
H	-0.30095600	0.71513900	1.68034000
O	-2.74272200	1.03346700	0.94041600
C	-3.89152800	0.51242000	0.14216700
O	-3.56547900	0.18951000	-1.06731000
O	-4.94566100	0.43345100	0.77820100
C	0.71469500	-1.47230400	0.26652300
C	-1.23650100	-1.07940500	-0.77548200
C	0.34964000	-2.64627600	-0.41745300
H	-2.20901900	-0.56773300	-1.08108700
H	-1.42664600	-2.97852000	-1.60782900
N	-0.86774200	-2.35027600	-1.05195200
N	-0.30057600	-0.52665900	0.01627600
C	0.89797200	1.62780300	0.13513300
C	1.58731700	2.43071000	1.05732900
C	1.30786700	1.61168000	-1.20811300
C	2.67811700	3.20873800	0.64395300
H	1.26879000	2.45361000	2.09628100
C	2.40002700	2.38490300	-1.62250900
H	0.77798900	0.99755200	-1.93118500
C	3.08846900	3.18520900	-0.69739900
H	3.20354100	3.82855400	1.36241500
H	2.71087000	2.36764700	-2.66156700
H	3.93279900	3.78528900	-1.01897300
C	1.89251500	-1.39863300	1.01951300
H	2.18467300	-0.49069100	1.53202200
C	2.68393300	-2.54918300	1.06349300
H	3.60519400	-2.53751700	1.63417500
C	1.13940200	-3.80053100	-0.37805300
H	0.85855000	-4.70635800	-0.90174400

C	2.31379700	-3.73110000	0.37687300
H	2.95749900	-4.60093500	0.43869900

TS2 Bz-HIm LS

C	-1.00202400	-1.38769800	-0.57105100
H	-0.74409800	-0.88205100	-1.49068000
H	-1.13711700	-2.46209500	-0.61775300
C	-1.50506200	-0.60923700	0.66920900
H	-1.00717200	-1.00181600	1.55866900
O	-2.90955000	-0.86932800	0.90241500
C	-3.65453400	-1.16712400	-0.30568700
O	-2.84486200	-1.29388800	-1.32593200
O	-4.87795400	-1.27337300	-0.20952600
C	1.84270100	-0.61549800	-0.33120000
C	1.34202000	-2.56746500	0.55329900
C	3.04791100	-1.17620700	0.15050800
H	0.79597900	-3.43461900	0.88714200
H	3.31408100	-3.07619500	1.13820300
N	2.69190900	-2.41190500	0.70640600
N	0.80229500	-1.52179700	-0.05689100
C	-1.20480700	0.87412900	0.54383200
C	-0.37971300	1.50493900	1.48538200
C	-1.72592400	1.62229500	-0.52879000
C	-0.05648700	2.86417500	1.35582100
H	0.01304400	0.93495400	2.32337800
C	-1.40573300	2.97951400	-0.65936900
H	-2.37664700	1.13959300	-1.25189500
C	-0.56522000	3.60377400	0.27881900
H	0.58312700	3.34172700	2.09063200
H	-1.81652100	3.55156200	-1.48473600
H	-0.32208700	4.65642100	0.17701300
C	1.83014700	0.64429400	-0.95279500
H	0.90910100	1.09541800	-1.30195700
C	4.27953800	-0.51546100	0.03276100
C	3.05218600	1.30337900	-1.07463400
H	3.08133600	2.28057500	-1.54218900
C	4.25817800	0.73308900	-0.59056600
H	5.18466200	1.28443700	-0.70539500
H	5.20276200	-0.94468000	0.40406700

TS2 Bz-HIm MS

C	-0.53753700	-1.58715800	-0.83294800
H	-0.17870400	-1.10562500	-1.74480600
H	0.15757500	-2.38679800	-0.55196300
C	-0.62034100	-0.57519000	0.32302300
H	-0.21125300	-0.83820500	1.28802600
O	-1.82402800	-2.13784200	-1.16563100
C	-2.64209300	-2.38427100	0.01977900
O	-2.08806000	-1.85325600	1.07425100
O	-3.69150900	-3.01008200	-0.14461600
C	2.29677900	-0.12083400	0.08418000
C	1.10487000	1.58988500	-0.61709300
C	3.20960400	0.87663800	-0.32934700
H	0.27045800	2.22542800	-0.86719700
H	2.74626100	2.82745200	-1.12744400

N	2.41779600	1.94530300	-0.76878600
N	0.99510400	0.36752500	-0.11651900
C	-1.55635100	0.58774200	0.27160100
C	-1.71747700	1.38862400	1.41549100
C	-2.24311400	0.92218200	-0.90907000
C	-2.54895700	2.51322900	1.38311800
H	-1.20881300	1.11341200	2.33420300
C	-3.07779400	2.04864000	-0.94085200
H	-2.16616100	0.28434000	-1.78288600
C	-3.22893600	2.84992200	0.20048700
H	-2.67947800	3.11630600	2.27526400
H	-3.62174000	2.28799500	-1.84826700
H	-3.88284500	3.71529700	0.17617700
C	2.74988600	-1.34503800	0.60135800
H	2.05854200	-2.11244700	0.92847600
C	4.59760300	0.69471100	-0.25206400
C	5.03937600	-0.52676900	0.26015100
H	6.10405500	-0.71489600	0.33962300
C	4.12992200	-1.53037100	0.68228200
H	4.51970300	-2.46188400	1.07596000
H	5.29823700	1.45761400	-0.57063200

TS1 2I-1M-Im LS

C	-0.89551000	0.82050400	1.28322900
H	-1.43502100	0.40921400	2.12768200
H	-0.18898600	1.62364300	1.45469800
C	-1.55067100	0.74518500	-0.04646600
H	-0.91780500	1.09671900	-0.86760400
O	-2.34690000	1.79511000	0.54230200
C	-1.78946000	3.66727300	-0.02910600
O	-0.71116600	3.46332200	-0.54655800
O	-2.71969100	4.31444800	0.36451900
C	0.16248000	-1.93945000	1.76067200
C	1.55894000	-0.75343800	0.56207700
C	1.08733200	-2.81883700	1.24243000
H	-0.69913400	-2.13701000	2.37368900
H	1.18134200	-3.88640500	1.33722700
N	1.97244200	-2.06118700	0.47978200
N	0.47599800	-0.65617200	1.33408400
C	-2.27230200	-0.53354600	-0.37567100
C	-1.66231200	-1.49546800	-1.19885300
C	-3.53580700	-0.79569300	0.17692500
C	-2.29953100	-2.71801900	-1.45380200
H	-0.69378800	-1.28300300	-1.64680700
C	-4.17584000	-2.01736800	-0.07922000
H	-4.00269600	-0.02396500	0.78039300
C	-3.55789600	-2.98350500	-0.88984300
H	-1.82566600	-3.45483600	-2.09502900
H	-5.15657600	-2.21248200	0.34277200
H	-4.05681900	-3.92624100	-1.09079600
I	2.48116900	0.84416400	-0.39675800
C	3.12122900	-2.57342200	-0.26879800
H	3.00819200	-2.36030900	-1.33429900
H	4.04649200	-2.11970300	0.09364300
H	3.17756800	-3.65311300	-0.12799200

TS1 2I-1M-Im MS

C	1.00914500	1.18979700	-1.39628500
H	1.35836300	0.73287600	-2.32263400
H	0.00318000	1.60175800	-1.48846400
C	1.24609000	0.45073900	-0.14302600
H	0.72028100	0.85279300	0.71685600
O	1.96691100	2.13784300	-0.84701800
C	1.22598700	3.54984000	-0.03195600
O	0.03185600	3.28787100	0.13660300
O	2.13051600	4.34456800	0.12948100
C	0.08444000	-2.47291300	-0.52893900
C	-1.54459200	-1.06801100	-0.12427500
C	-1.08941700	-3.19137600	-0.61112500
H	1.10225300	-2.80117300	-0.65592800
H	-1.27271200	-4.23051300	-0.82275400
N	-2.12328700	-2.29453000	-0.35221300
N	-0.21776100	-1.15278500	-0.22519700
C	2.42117800	-0.40766000	0.10260300
C	2.58866300	-0.96832900	1.38288200
C	3.35988400	-0.69609100	-0.90467100
C	3.66720800	-1.81781000	1.65161300
H	1.86675600	-0.74133500	2.16170200
C	4.44354000	-1.54070500	-0.63518700
H	3.26862500	-0.23357900	-1.88061000
C	4.59723600	-2.10759000	0.64007500
H	3.78923600	-2.24439000	2.64114200
H	5.17355000	-1.74621400	-1.41047300
H	5.44033400	-2.75820400	0.84737300
I	-2.58674000	0.67470800	0.32866000
C	-3.55243100	-2.60880700	-0.32667900
H	-3.97188300	-2.40243900	0.66069700
H	-4.08662300	-2.01888500	-1.07499100
H	-3.68265600	-3.66779800	-0.55173800

Intermediate 1 2I-1M-Im LS

C	-0.48623300	0.55104800	1.18856300
H	-1.15188600	0.50099500	2.05276900
H	0.28003100	1.31517300	1.34056600
C	-1.29701400	0.96294300	-0.07610700
H	-0.60138800	0.98103500	-0.92663400
O	-1.81489300	2.26365300	0.18472800
C	-0.78820200	3.40347200	0.06834700
O	0.38824200	2.97548100	-0.16527400
O	-1.31252400	4.51123900	0.24400400
C	-0.42676100	-1.97889200	1.38014400
C	1.33719700	-1.00947700	0.41102500
C	0.42476800	-2.97463300	0.98268100
H	-1.39357600	-2.02404600	1.84877600
H	0.33852500	-4.04281400	1.06741000
N	1.51963200	-2.35909200	0.37793000
N	0.15736800	-0.76897000	1.02901900
C	-2.42879000	-0.01121400	-0.32580100
C	-2.25056000	-1.10634800	-1.18724100
C	-3.65247000	0.14122700	0.34942600

C	-3.27090200	-2.05554400	-1.35736200
H	-1.32142400	-1.20608500	-1.74480600
C	-4.67220600	-0.80578200	0.18248500
H	-3.79607600	1.02153900	0.96670700
C	-4.48264900	-1.91094600	-0.66525700
H	-3.12852400	-2.89088200	-2.03585000
H	-5.61857800	-0.67589200	0.69750300
H	-5.27768100	-2.63727400	-0.79997400
I	2.61962800	0.42147400	-0.33869700
C	2.67870100	-3.05325700	-0.19461800
H	2.77807100	-2.80939800	-1.25410500
H	3.59031700	-2.76548000	0.33269300
H	2.52702000	-4.12713300	-0.08813500

Intermediate 1 2I-1M-Im MS

C	0.27505500	-2.06376100	-1.02964600
H	-0.27279600	-2.91138500	-1.45172900
H	0.24556000	-1.21252600	-1.71870200
C	-0.37133600	-1.69647100	0.33149900
H	-0.29509800	-2.58897000	0.96207900
O	1.62099200	-2.49906200	-0.81051700
C	2.71191200	-1.47543600	-0.96754900
O	2.28898700	-0.30285000	-1.25987900
O	3.84017900	-1.94736300	-0.74426800
C	1.53600100	-1.11249500	1.86241600
C	0.75412600	0.57594400	0.63326800
C	2.38152800	-0.05484800	2.02554200
H	1.60590900	-2.12268200	2.22121100
H	3.30893700	0.01991500	2.56245500
N	1.87326000	0.99671800	1.26639600
N	0.51403000	-0.70504400	1.00619200
C	-1.82602100	-1.24748400	0.30961100
C	-2.61150600	-1.34233700	-0.84961200
C	-2.41042700	-0.75934600	1.49315100
C	-3.95653700	-0.94430700	-0.82894200
H	-2.18395200	-1.70906700	-1.77580800
C	-3.75080100	-0.35793600	1.51483600
H	-1.81268000	-0.68605900	2.39794800
C	-4.52953300	-0.44792500	0.34992900
H	-4.55218300	-1.01901400	-1.73245200
H	-4.18758300	0.02147300	2.43249700
H	-5.56909000	-0.13843500	0.36351300
C	2.63160600	2.20529200	0.92519000
H	3.46201500	2.30193900	1.62447300
H	3.01486900	2.08136800	-0.09124700
H	1.99971400	3.09219400	0.99567500
I	-0.34868500	1.75755500	-0.65658200

TS2 2I-1M-Im LS

C	-0.67225700	0.80252000	0.99530000
H	-1.15017700	0.37681900	1.86658900
H	0.11995200	1.52258000	1.16655200
C	-1.31731300	0.69801400	-0.40828000
H	-0.53539400	0.46972700	-1.13779600

O	-1.84210000	1.97483000	-0.83787200
C	-2.25660900	2.83781500	0.25336100
O	-1.91281700	2.32352700	1.40485400
O	-2.82861800	3.88864400	-0.04146500
C	0.09114100	-1.94606000	1.20995300
C	1.71876800	-0.72568600	0.37139000
C	1.15296500	-2.79120200	0.98342900
H	-0.89394000	-2.15716700	1.58838600
H	1.25966800	-3.84958400	1.14357800
N	2.17994000	-2.01372400	0.45221800
N	0.46397000	-0.66679700	0.82543900
C	-2.36937000	-0.39850700	-0.44383200
C	-2.23427600	-1.47456600	-1.33282100
C	-3.47907400	-0.35341600	0.42121900
C	-3.18718900	-2.50485200	-1.35850100
H	-1.38826200	-1.50424700	-2.01499900
C	-4.43193400	-1.37948500	0.39608700
H	-3.59346700	0.48650200	1.10050300
C	-4.28780600	-2.46030900	-0.49066700
H	-3.07677300	-3.32931200	-2.05551600
H	-5.28938500	-1.33334600	1.05931600
H	-5.03083200	-3.25095200	-0.51124400
C	3.50677700	-2.49557900	0.05840500
H	4.28250900	-1.99275000	0.63994900
H	3.55928400	-3.56742200	0.24968800
H	3.67644000	-2.31612300	-1.00550300
I	2.80789300	0.88416600	-0.36165100

TS2 2I-1M-Im MS

C	-0.87519500	-1.19201900	-1.27381600
H	-0.75158700	-0.50720100	-2.11633800
H	-0.01311400	-1.86822400	-1.22788100
C	-0.95179700	-0.40904900	0.04839800
H	-0.39406700	-0.76634600	0.90297700
O	-2.07701000	-1.92996500	-1.55375400
C	-2.66232000	-2.53010700	-0.35196500
O	-2.07775700	-2.07713100	0.71826100
O	-3.59111900	-3.32312600	-0.52776100
C	0.20033100	2.10619400	-0.94350000
C	1.73612500	0.69217000	-0.25126200
C	1.42146900	2.70764900	-1.14261800
H	-0.79330500	2.47315300	-1.13482500
H	1.67523700	3.67238000	-1.54580700
N	2.39041500	1.80945000	-0.70197400
N	0.41707000	0.85308200	-0.38972500
C	-2.06317900	0.54920700	0.31845300
C	-2.14169300	1.16880400	1.57743900
C	-3.01390500	0.86407300	-0.66874500
C	-3.15234900	2.09634100	1.84980100
H	-1.42080000	0.90688300	2.34619700
C	-4.02820600	1.79186500	-0.39476000
H	-2.99342800	0.35335500	-1.62564100
C	-4.09795100	2.41374700	0.86046400
H	-3.21354200	2.56076500	2.82820200
H	-4.77117800	2.01473400	-1.15290400

H	-4.88838200	3.12604100	1.07277300
C	3.83896700	2.03082200	-0.72308700
H	4.03315900	3.01880500	-1.14076200
H	4.24725600	1.98612100	0.28886600
H	4.32948400	1.27976600	-1.34610600
I	2.67938000	-0.98083700	0.54661100

ZnI_2SO_2 complex

Zn	-1.59824100	0.00015700	-0.00011400
I	-2.53512000	2.43044800	-0.02044100
I	-2.53789800	-2.42909200	0.02053500
O	-0.10768000	0.08345800	1.45208300
O	-0.10799500	-0.08483300	-1.45230600
C	0.03393700	1.04579600	2.59072000
H	0.26424900	0.55170400	3.52751500
H	-0.72541900	1.82099400	2.60294500
C	1.05496800	1.03849200	1.50487400
H	0.98262000	1.82250900	0.75485700
C	0.03384000	-1.04762900	-2.59048400
H	0.26417800	-0.55390200	-3.52746600
H	-0.72543600	-1.82290600	-2.60246200
C	1.05478700	-1.03968800	-1.50454700
H	0.98250900	-1.82338500	-0.75419200
C	2.38915800	-0.40178900	-1.67925900
C	3.54394000	-1.14262300	-1.38319400
C	2.50453500	0.91230400	-2.16434300
C	4.80996500	-0.58114300	-1.59189400
H	3.45727900	-2.14839300	-0.98283100
C	3.77204500	1.47506700	-2.36181000
H	1.60662500	1.48829800	-2.36696600
C	4.92723300	0.72735500	-2.08344900
H	5.69870700	-1.15725800	-1.35884400
H	3.85907100	2.48991800	-2.73515800
H	5.90804500	1.16277000	-2.24243000
C	2.38940200	0.40068800	1.67939800
C	3.54408800	1.14157800	1.38310900
C	2.50493700	-0.91338900	2.16449700
C	4.81018100	0.58015600	1.59155900
H	3.45728200	2.14733500	0.98274300
C	3.77250800	-1.47609100	2.36172400
H	1.60709400	-1.48942800	2.36729100
C	4.92760500	-0.72833400	2.08309900
H	5.69885200	1.15629900	1.35831200
H	3.85966100	-2.49093200	2.73507100
H	5.90846800	-1.16370600	2.24188400

TS ZnI_2 + SO + CO_2

Zn	-1.39337600	0.02345000	-0.36190700
I	-2.84045800	-1.66947200	1.06783600
I	-2.13619900	2.26985600	-1.51311300
O	-0.29398100	-1.24889000	-1.56703400
O	0.18535500	0.51394000	0.75685000
C	-0.47624200	-2.66092900	-2.01763800
H	-0.14052600	-2.81971000	-3.03611400
H	-1.42937000	-3.09377100	-1.73156000

C	0.52676400	-2.35707200	-0.95761800
H	0.22900700	-2.57948100	0.06404500
C	0.01900500	2.15406900	2.20069300
H	0.54675800	2.52140100	3.07841500
H	-1.06736900	2.08728800	2.23615300
C	0.66966000	1.84712200	0.91487000
H	0.21959900	2.52395800	0.14919900
C	2.17946700	1.98060900	0.87917300
C	2.76870400	3.23697700	1.09916700
C	2.98385300	0.86329000	0.61839100
C	4.16324000	3.37171500	1.07688300
H	2.14670500	4.11402200	1.26808300
C	4.37951100	1.00138800	0.58967200
H	2.51696400	-0.09593800	0.42772000
C	4.97137000	2.25133700	0.82223400
H	4.61655900	4.34282500	1.24418700
H	4.99435400	0.13284300	0.37979500
H	6.05084500	2.35666200	0.79762700
C	1.99064600	-2.26676000	-1.20488600
C	2.87150700	-2.89539100	-0.30882700
C	2.50190000	-1.55121300	-2.30145500
C	4.25595300	-2.82821000	-0.52017600
H	2.47901700	-3.42881800	0.55113100
C	3.88514000	-1.47808000	-2.50575300
H	1.82024100	-1.02647600	-2.96306600
C	4.76510500	-2.12130700	-1.62020200
H	4.93126900	-3.32114900	0.17098700
H	4.27651100	-0.91614300	-3.34665600
H	5.83630000	-2.06639400	-1.78357600
C	0.60500100	-0.74093500	2.79781400
O	0.35184700	0.26614100	3.39967300
O	0.90349000	-1.79302300	2.35756900

ZnI_2(SC)(SO) complex

Zn	1.95088100	-0.19214800	-0.19912100
I	0.98253300	-2.45082100	0.78808400
I	4.33139100	0.79428900	-0.27280800
O	0.81777900	1.23826700	0.82825800
O	-1.29063300	0.17670900	-1.01892100
C	0.86972000	1.56730300	2.28492000
H	0.98865000	2.62764900	2.47525700
H	1.48643600	0.89124900	2.86738100
C	-0.40228100	1.06849000	1.68488600
H	-0.64640100	0.02133800	1.84551100
C	-2.51717200	0.05400100	-3.01986300
H	-2.80623100	-0.96674000	-3.27125000
H	-3.01479500	0.77464500	-3.66364500
C	-2.67721300	0.35963700	-1.50322500
H	-2.93020000	1.40830700	-1.32327000
C	-3.61220000	-0.56244100	-0.76603500
C	-4.96994200	-0.21701300	-0.67236100
C	-3.14792300	-1.75692200	-0.19319300
C	-5.86813100	-1.07192800	-0.02010200
H	-5.32403600	0.72112300	-1.09170200
C	-4.04756200	-2.60432100	0.46930900

H	-2.09369300	-2.01463400	-0.23338400
C	-5.40723600	-2.26770700	0.55341600
H	-6.91688300	-0.80394300	0.04849600
H	-3.68254600	-3.52051000	0.91984600
H	-6.09981300	-2.92698900	1.06537800
C	-1.54307300	1.96332200	1.34661200
C	-2.83874800	1.59661400	1.74217700
C	-1.34493700	3.15166500	0.62088000
C	-3.93131200	2.41796700	1.42803700
H	-3.00072800	0.66597100	2.27760000
C	-2.43803100	3.96646300	0.29864600
H	-0.34528800	3.41482800	0.28965900
C	-3.73457800	3.60342000	0.70426500
H	-4.92844700	2.12550200	1.73868400
H	-2.28133100	4.88155700	-0.26242300
H	-4.57861900	4.24046700	0.46202800
C	-0.41791100	0.14691400	-2.05717200
O	-1.05961000	0.17940600	-3.24999200
O	0.80505400	0.09662000	-1.92026500

TS3

Zn	1.83056000	0.14804000	-0.05570300
I	3.94222100	-0.92681000	-1.15093300
I	1.50686300	2.33717900	1.44445400
O	1.04705900	-1.38998900	1.13675000
O	0.20160600	0.08739500	-1.23547800
C	1.51015400	-2.79683900	1.33909300
H	1.45216300	-3.10970900	2.37532600
H	2.41486200	-3.03787400	0.79000600
C	0.24276500	-2.53270900	0.59642000
H	0.27998000	-2.59122000	-0.48849800
C	-0.77659100	1.56586100	-1.53029100
H	-0.99969700	1.50639200	-2.58714200
H	-0.12478200	2.34344400	-1.15539100
C	-1.11299700	0.41652900	-0.67074000
H	-1.09491800	0.61735400	0.40327700
C	-2.22961100	-0.49136600	-1.08991500
C	-3.44045600	-0.47790000	-0.38054200
C	-2.08513500	-1.32741100	-2.20857400
C	-4.50807500	-1.28453900	-0.79431500
H	-3.54340100	0.15098800	0.49916100
C	-3.15129800	-2.14080400	-2.61812000
H	-1.13516200	-1.33718000	-2.73409800
C	-4.36650200	-2.11753400	-1.91524500
H	-5.43880900	-1.27598600	-0.23681300
H	-3.03610800	-2.78860600	-3.48116900
H	-5.19143600	-2.74687900	-2.23335700
C	-1.09863800	-2.71059600	1.22116200
C	-2.01178200	-3.60421200	0.63941300
C	-1.45245300	-2.00299800	2.38181900
C	-3.26729200	-3.80507800	1.22575000
H	-1.75072800	-4.12958700	-0.27446300
C	-2.71344900	-2.19918400	2.96128100
H	-0.74862700	-1.29515400	2.80836300
C	-3.62091100	-3.10379800	2.38868600

H	-3.97024100	-4.49312100	0.76881600
H	-2.98544000	-1.64995100	3.85662000
H	-4.59524000	-3.25711700	2.84088000
C	-3.74316500	2.93677200	-1.53502200
C	-2.28406600	3.79914800	-0.14480200
C	-4.43754600	3.83015300	-0.74496400
H	-4.10805700	2.30280600	-2.32416600
N	-3.49909700	4.36811500	0.13108200
H	-1.37306100	4.01238000	0.39178300
H	-5.47538400	4.11240600	-0.73353000
H	-3.67686200	5.05623900	0.84505700
N	-2.40438200	2.93465700	-1.15292700

Intermediate 2

Zn	1.75001800	0.00292900	-0.17723400
I	3.99277000	-1.11147600	-0.94536000
I	1.55352300	2.36434600	1.22792200
O	0.96338200	-1.37010400	1.24052900
O	0.18310600	-0.15366300	-1.29493400
C	1.32274600	-2.79690100	1.49510100
H	1.23187300	-3.07331500	2.53963400
H	2.21532500	-3.12047400	0.96890700
C	0.08766900	-2.46819000	0.72288400
H	0.13168200	-2.54981600	-0.35960300
C	-1.07380600	1.83048600	-1.63725500
H	-1.30172300	1.68566700	-2.69504700
H	-0.09594300	2.30440800	-1.53339700
C	-1.01829400	0.45461300	-0.90112000
H	-1.05695900	0.67792000	0.18545700
C	-2.24197400	-0.38254600	-1.24990100
C	-3.43036300	-0.27741200	-0.50929900
C	-2.18930600	-1.25803800	-2.34721200
C	-4.56532400	-1.01680600	-0.87364300
H	-3.45803600	0.34839800	0.38036300
C	-3.32079400	-2.00148900	-2.71150200
H	-1.24727100	-1.35284100	-2.87781400
C	-4.51500100	-1.87727600	-1.98048200
H	-5.47159500	-0.94375700	-0.28087200
H	-3.27290400	-2.67946000	-3.55801100
H	-5.38885900	-2.45684500	-2.26094600
C	-1.27562200	-2.52649000	1.32141000
C	-2.24381800	-3.35845400	0.73664000
C	-1.60295500	-1.76074700	2.45261000
C	-3.52538200	-3.44567000	1.29367600
H	-2.00278800	-3.92320800	-0.15901100
C	-2.88990500	-1.84220200	3.00295200
H	-0.85483400	-1.09898400	2.87759400
C	-3.85168800	-2.68880000	2.42955000
H	-4.26893700	-4.08749900	0.83355500
H	-3.13950200	-1.25090000	3.87821200
H	-4.84567100	-2.75526400	2.86021700
C	-3.40774500	2.88568400	-1.46211900
C	-1.88841800	3.43937500	0.07350700
C	-4.01776300	3.72874600	-0.56880200
H	-3.79640500	2.37749000	-2.32627200

N	-3.04994300	4.05937400	0.37967800
H	-0.95075800	3.47015500	0.62074600
H	-5.02532900	4.10021400	-0.53131100
H	-3.18015300	4.66452700	1.17597100
N	-2.08730300	2.73155100	-1.04880100

TS4

Zn	-1.77428600	0.05204600	0.17578300
I	-3.68094900	-1.76402400	0.28959800
I	-1.80345900	2.47438000	-1.11297100
O	-0.56789500	-0.88845800	-1.39952700
O	-0.13909800	0.08788700	1.30297600
C	-1.01797400	-1.97880100	-2.31698300
H	-0.65623300	-1.85796400	-3.33201600
H	-2.05975100	-2.25396100	-2.18942700
C	-0.04742400	-2.29368400	-1.23247400
H	-0.44728100	-2.77064000	-0.34153100
C	1.29315000	2.02127900	1.27220900
H	1.66165300	2.06556100	2.29757400
H	0.32404800	2.52211000	1.20833900
C	1.10801200	0.54284100	0.80234500
H	1.06559800	0.55868900	-0.30014000
C	2.28993800	-0.31290000	1.22720100
C	3.45579600	-0.33773500	0.44288800
C	2.25046400	-1.04945300	2.42162200
C	4.58032700	-1.06455100	0.85669800
H	3.47612900	0.17861900	-0.51440200
C	3.37063400	-1.78675500	2.83139600
H	1.34356700	-1.04090700	3.01378700
C	4.54194300	-1.78978000	2.05651000
H	5.46773300	-1.08767500	0.23237100
H	3.33065100	-2.35457800	3.75533100
H	5.40731100	-2.36075600	2.37735600
C	1.40451600	-2.49104300	-1.49316500
C	2.11525200	-3.42915400	-0.72774200
C	2.06815900	-1.78531400	-2.51291500
C	3.46621700	-3.68443900	-0.99722600
H	1.61624600	-3.95432700	0.08101600
C	3.41910100	-2.03830000	-2.78036200
H	1.53071800	-1.03518200	-3.08624800
C	4.11942000	-2.99631300	-2.02937300
H	4.00688700	-4.40791000	-0.39704600
H	3.92315700	-1.49916800	-3.57629000
H	5.16424000	-3.19809500	-2.24152800
C	3.59020200	2.94851900	0.60904400
C	1.88586600	3.26676500	-0.79565900
C	4.07958200	3.61629900	-0.48409800
H	4.08445700	2.58967100	1.49418100
N	2.99788100	3.80175000	-1.34546900
H	0.88138200	3.23886100	-1.21004000
H	5.07301200	3.95885800	-0.70888200
H	3.02339000	4.26809500	-2.23964000
N	2.22872300	2.75165600	0.39539600
C	-0.95581000	0.73531500	2.96956100
O	-0.06334500	0.93560900	3.75637700

O	-2.10530900	0.71493700	2.58719400

Intermediate 3

Zn	-1.82790500	0.15971200	0.47579800
I	-4.03828000	-1.28301200	0.27084800
I	-1.60662800	2.59988400	-0.81589900
O	-0.72825100	-0.87877800	-1.08943600
O	0.29486100	0.07343100	1.19058300
C	-1.23753500	-1.59361100	-2.29591800
H	-0.62790800	-1.41307100	-3.17394600
H	-2.31300500	-1.52793900	-2.42346000
C	-0.70524100	-2.39615400	-1.16572300
H	-1.45144200	-2.81897800	-0.49613800
C	1.71415500	2.06259600	1.08301100
H	2.21906700	2.07096800	2.04814800
H	0.75644800	2.57988300	1.17675200
C	1.47867700	0.61709900	0.56826600
H	1.19395900	0.67402200	-0.48847400
C	2.72044100	-0.23747600	0.70928100
C	3.42997800	-0.60841400	-0.44343600
C	3.21166800	-0.60369700	1.97642800
C	4.63701700	-1.31460200	-0.33909500
H	3.02835300	-0.36634900	-1.42401100
C	4.41239100	-1.31875000	2.07909000
H	2.64706000	-0.33028400	2.86380500
C	5.13366900	-1.66637200	0.92400000
H	5.17067800	-1.60847000	-1.23662500
H	4.78512400	-1.60432400	3.05728900
H	6.06382400	-2.21885000	1.00877600
C	0.63929200	-3.03126200	-1.12396500
C	1.19418800	-3.32420000	0.13588100
C	1.34564300	-3.37703300	-2.28720000
C	2.43667300	-3.95833100	0.23091600
H	0.65559800	-3.04307200	1.03563700
C	2.59136600	-4.01651500	-2.19266400
H	0.91859500	-3.17563600	-3.26512100
C	3.13588000	-4.31041500	-0.93443600
H	2.86120500	-4.17029900	1.20577000
H	3.12545200	-4.29541500	-3.09512300
H	4.09719600	-4.80806400	-0.86022300
C	3.92258100	2.80135500	0.02641500
C	2.02960200	3.51749600	-0.91700100
C	4.26760300	3.55687000	-1.06394200
H	4.52954300	2.26345300	0.73329800
N	3.07045500	3.99069500	-1.63490300
H	0.96949400	3.64573900	-1.11235300
H	5.23228100	3.80757900	-1.46547300
H	2.97955600	4.56933400	-2.45642200
N	2.53101800	2.80011500	0.10033200
C	-0.15250000	0.43387800	2.57544000
O	0.70188100	0.72576300	3.42177600
O	-1.42691700	0.36130500	2.53352300

TS5

Zn	-2.38349300	0.17011900	0.24797200

I	-4.91528600	-0.36821900	0.42390900
I	-1.07861700	2.24739400	-0.89709300
O	-1.61226600	-1.41190400	-0.85310900
O	0.68475100	-0.41165000	0.89159400
C	-2.05980000	-2.81719600	-1.07649000
H	-2.09496700	-3.08462700	-2.12626300
H	-2.90523500	-3.10973700	-0.46287500
C	-0.73272500	-2.54055800	-0.44280100
H	-0.66434100	-2.64714100	0.63526200
C	1.99526400	1.53384500	1.55628000
H	2.67810100	1.64282000	2.38653700
H	1.35269800	2.36422400	1.29371300
C	1.98128200	0.24323800	0.70148900
H	1.99695900	0.50472400	-0.35491900
C	3.10737400	-0.70398100	1.04359100
C	4.09467400	-0.98943100	0.09233400
C	3.17363400	-1.29929800	2.31610800
C	5.14899900	-1.85969800	0.40508400
H	4.03166900	-0.54768000	-0.89767400
C	4.22181100	-2.17318900	2.62805900
H	2.40867200	-1.08180700	3.05623100
C	5.21408900	-2.45427400	1.67291700
H	5.90510200	-2.08155800	-0.34060900
H	4.26745200	-2.63392400	3.60911100
H	6.02561000	-3.13222000	1.91613900
C	0.55108200	-2.65250100	-1.19194200
C	1.51023800	-3.58888500	-0.77293500
C	0.80903100	-1.83724700	-2.30575500
C	2.70686900	-3.73727700	-1.48571300
H	1.32661500	-4.19769100	0.10760900
C	2.01078800	-1.98324200	-3.01381800
H	0.07901200	-1.08800400	-2.59437500
C	2.95718000	-2.93922500	-2.61243400
H	3.44376900	-4.46186500	-1.15658900
H	2.20390700	-1.35557000	-3.87776900
H	3.88314000	-3.05612900	-3.16626200
C	4.77114900	2.40599600	0.36756600
C	3.00422600	3.23763300	-0.64404800
C	5.24042900	3.24527200	-0.62065600
H	5.32068600	1.80298200	1.06924100
N	4.11076400	3.76058200	-1.25032600
H	1.98466600	3.44962800	-0.92770400
H	6.24166700	3.50521200	-0.91471500
H	4.10283300	4.41088600	-2.02036200
N	3.38026900	2.41615600	0.33787700
C	-0.05534200	0.13624600	1.92891400
O	0.62022000	0.91816200	2.69689600
O	-1.28584300	-0.15672600	1.95444000

Intermediate 4

Zn	1.95088100	-0.19214800	-0.19912100
I	0.98253300	-2.45082100	0.78808400
I	4.33139100	0.79428900	-0.27280800
O	0.81777900	1.23826700	0.82825800
O	-1.29063300	0.17670900	-1.01892100

C	0.86972000	1.56730300	2.28492000
H	0.98865000	2.62764900	2.47525700
H	1.48643600	0.89124900	2.86738100
C	-0.40228100	1.06849000	1.68488600
H	-0.64640100	0.02133800	1.84551100
C	-2.51717200	0.05400100	-3.01986300
H	-2.80623100	-0.96674000	-3.27125000
H	-3.01479500	0.77464500	-3.66364500
C	-2.67721300	0.35963700	-1.50322500
H	-2.93020000	1.40830700	-1.32327000
C	-3.61220000	-0.56244100	-0.76603500
C	-4.96994200	-0.21701300	-0.67236100
C	-3.14792300	-1.75692200	-0.19319300
C	-5.86813100	-1.07192800	-0.02010200
H	-5.32403600	0.72112300	-1.09170200
C	-4.04756200	-2.60432100	0.46930900
H	-2.09369300	-2.01463400	-0.23338400
C	-5.40723600	-2.26770700	0.55341600
H	-6.91688300	-0.80394300	0.04849600
H	-3.68254600	-3.52051000	0.91984600
H	-6.09981300	-2.92698900	1.06537800
C	-1.54307300	1.96332200	1.34661200
C	-2.83874800	1.59661400	1.74217700
C	-1.34493700	3.15166500	0.62088000
C	-3.93131200	2.41796700	1.42803700
H	-3.00072800	0.66597100	2.27760000
C	-2.43803100	3.96646300	0.29864600
H	-0.34528800	3.41482800	0.28965900
C	-3.73457800	3.60342000	0.70426500
H	-4.92844700	2.12550200	1.73868400
H	-2.28133100	4.88155700	-0.26242300
H	-4.57861900	4.24046700	0.46202800
C	-0.41791100	0.14691400	-2.05717200
O	-1.05961000	0.17940600	-3.24999200
O	0.80505400	0.09662000	-1.92026500
